# Relationships Among Arsenic-Related Traits, Including Rice Grain Arsenic Concentration and Straighthead Resistance, as Revealed by Genome-Wide Association

**DOI:** 10.3389/fgene.2021.787767

**Published:** 2022-03-14

**Authors:** Shannon R. M. Pinson, D. Jo Heuschele, Jeremy D. Edwards, Aaron K. Jackson, Santosh Sharma, Jinyoung Y. Barnaby

**Affiliations:** ^1^ Dale Bumpers National Rice Research Center, United States Department of Agriculture—Agricultural Research Service, Stuttgart, AR, United States; ^2^ Plant Science Research Unit, United States Department of Agriculture—Agricultural Research Service, St. Paul, CO, United States

**Keywords:** rice, arsenic, straighthead disorder, genome-wide association, bayesian network, QTL

## Abstract

There is global concern that rice grains and foods can contain harmful amounts of arsenic (As), motivating breeders to produce cultivars that restrict As accumulation in grains to protect human health. Arsenic is also toxic to plants, with straighthead disorder (StHD), causing panicle sterility, being observed in rice. The genetic variation in StHD resistance suggests that plants have evolved mechanisms that reduce As toxicity, possibly *via* regulation of As uptake, transport, or detoxification/sequestration. Because these mechanisms could also underlie the wide (3- to 100-fold) differences in grain As concentration (grain-As) observed among diverse rice genotypes, it was hypothesized that some genes reduce both grain-As content and StHD susceptibility and may be detectable as co-located StDH and As quantitative trait loci (QTL). We used a machine-learning Bayesian network approach plus high-resolution genome-wide association study (GWAS) to identify QTL for grain-As and StHD resistance within the USDA Rice Minicore Collection (RMC). Arsenic enters roots through phosphorus (P) and silica (Si) transporters, As detoxification involves sulfur (S), and cell signaling to activate stress tolerance mechanisms is impacted by Si, calcium (Ca), and copper (Cu). Therefore, concentrations of Si, P, S, Ca, and Cu were included in this study to elucidate physiological mechanisms underlying grain-As and StHD QTL. Multiple QTL (from 9 to 33) were identified for each of the investigated As-associated traits. Although the QTL for StHD, Si, and grain-As did not overlap as heavily as our hypothesis predicted (4/33 StHD and 4/15 As QTL co-located), they do provide useful guidance to future research. Furthermore, these are the first StHD and Si QTL to be identified using high-density mapping, resulting in their being mapped to shorter, more precise genomic regions than previously reported QTL. The candidate genes identified provide guidance for future research, such as gene editing or mutation studies to further investigate the role of antioxidants and ROS scavenging to StHD resistance, as indicated by candidate genes around the commonly reported qStHD8-2 QTL. Other genes indicated for future study for improving grain-As and StHD include several multidrug and toxic compound extrusion (MATE) genes, F-box genes, and NIPs not documented to date to transport As.

## 1 Introduction

Arsenic (As) is naturally present in the environment, and trace amounts are found in all soil and groundwater, but it is toxic to animal and plant life and has been associated with various cancers, cardiovascular disease, and diabetes in humans (WHO/FAO Joint Expert Committee on Food Additives, 2010; FAO/WHO, 2013). The environmental occurrence of As concentrations differs geographically around the world due to different As contents of bedrock, and from manmade causes such as mining or use of arsenical solutions for tanning hides, for preserving wood, or as agricultural herbicides (Agency for Toxic Substances and Disease Registry, 2007). Plants acquire As through roots along with beneficial soil nutrients and accumulate it in vegetative tissues and seeds. While trace amounts of As may be expected in any plant product, concentrations of As in edible seeds is of higher concern in rice than other grains (Williams et al., 2007) primarily because rice is commonly grown in flooded paddies where the anaerobic soil conditions increase the availability of As (see reviews Abedi and Mojiri, 2020; Tang and Zhao, 2020b). Although rice produced in the United States has been shown safe for consumption by the general population, not all US Rice meets the more stringent requirements set for baby food (US Food and Drug Administration, 2016; US Food and Drug Administration, 2020). There is global interest in identifying and implementing strategies for reducing rice grain As concentrations (grain-As). In aerobic soils, As is predominantly present as arsenate where, like its chemical analogue phosphate, it is largely bound to metal oxides and unavailable for plant uptake. Under anaerobic (flooded) conditions, arsenate becomes chemically reduced to arsenite and is more available for plant uptake (Dixit and Hering, 2003). Because rice roots exude oxygen into the rhizosphere, some arsenate and phosphate remain available for plant uptake in flooded fields (Seyfferth et al., 2010). Arsenate enters plant roots through phosphate transporters (Meharg and Hartley-Whitaker, 2002; Cao et al., 2017; Ye et al., 2017), while arsenite travels into and through plants *via* Si transporters (Ma et al., 2008). Some soil microbes methylate inorganic As (i.e., arsenate and arsenite), converting it into less toxic organic As (oAs) forms such as dimethylarsinic acid (DMA), which also enters roots through the Lsi1 silicon transporter (Ma et al., 2008; Abedi and Mojiri, 2020; Tang and Zhao, 2020b). The predominant forms of As in rice grains are arsenite and DMA (Meharg et al., 2008; Meharg et al., 2009; Heitkemper et al., 2009).

One method proposed for reducing the amount of As in rice plants and grains is to produce the crop without a flood for part or all of its production period, production systems known in the United States as alternate wetting and drying (AWD) and furrow irrigation, respectively. Use of flooded paddies for rice production became preferred over millennia, however, because the flood water protects rice plants from weeds, insects, drought, temperature extremes, and some diseases, including rice blast. Because roots uptake As through phosphorus (P) and silica (Si) transporters, application of P (Begum et al., 2008; Choudhury et al., 2011) and Si fertilizers has also been evaluated as a mitigation strategy (Seyfferth and Fendorf, 2012; Matsumoto et al., 2015; Seyfferth et al., 2016, Seyfferth et al., 2018). Our research focuses instead on identifying rice genes and physiological factors that can reduce grain-As in a variety of production systems.

As exposure is known to reduce root and shoot growth (Heuschele et al., 2017; Kalita et al., 2018; Murugaiyan et al., 2019) and has long been associated with a physiological disorder in rice known as straighthead (StHD), so called because it is characterized by erect seed heads (panicles) upon maturity from poor seed set in often distorted spikelets. While direct evidence documenting the oAs DMA as the cause of StHD was only recently determined (Tang et al., 2020a), herbicides containing the synthetic oAs monosodium methanearsonate (MSMA) have been used for decades to induce StHD for the purpose of facilitating selection of StHD-resistant breeding progeny (Yan et al., 2005). Plant genes and mechanisms that reduce As uptake or enhance As detoxification by chelation and/or vacuolar sequestration would be expected to reduce both grain-As and the severity of As-induced plant stress, such as StHD.

Altering the Lsi1 transporter to reduce the root uptake of As may seem enticing, but alterations in this protein have been detrimental, substantially decreasing plant growth and grain yield as well as grain-As (Ma et al., 2008). Furthermore, of the quantitative trait loci (QTL) for grain-As reported to date (Norton et al., 2010; Norton et al., 2012a; Norton et al., 2014; Zhang et al., 2014; Yang et al., 2018; Frouin et al., 2019; Norton et al., 2019; Fernández-Baca et al., 2021; Liu et al., 2021), none have encompassed the Lsi1 locus, indicating that the wide natural variation observed for grain-As (3- to 100-fold differences reported by Norton et al. (2012b), Pinson et al. (2015), and Duan et al. (2017) is not caused by mutation in the Lsi1 gene. In contrast, grain-As QTL have co-located with the Lsi2 gene (Norton et al., 2019), which impacts the root-to-shoot transfer of Si and As (Ma et al., 2008), and to the ABCC1 gene (Norton et al., 2019) which reduces transport of As to grains by increasing vacuolar sequestration (Song et al., 2014). Heuschele et al. (2017) compared three rice cultivars with high grain-As with three low grain-As cultivars to see if their seedlings differed metabolically in response to arsenite. Data indicated that reduced grain-As concentrations were neither due to reduced root uptake (e.g., Lsi1) nor due to root-to-shoot transfer differences, but were instead associated with an increase in cysteine and glutathione (GSH) in the leaves, with cysteine being a key substrate for synthesis of GSH. With other studies showing that binding of As to either GSH or GSH-containing phytochelatins is required before As can be transported into vacuoles (Raab et al., 2004, Raab et al., 2005; Hossain et al., 2012; Song et al., 2014), there is a growing body of evidence on the importance of post-uptake metabolism in regulating grain-As. Because cysteine, GSH, and phytochelatins are sulfur (S)-based compounds, this further suggests that plants require sufficient sulfur to limit the accumulation of As in grains.

GSH is important not only for detoxification but also for stress tolerance. Like other causes of abiotic stress, As toxicity induces the production of reactive oxygen species (ROS) (Tripathi et al., 2013; Nath et al., 2014) which are themselves injurious to plants. The increased ability to scavenge ROS with antioxidants has been shown to reduce cell damage due to drought (Zhu et al., 2020; Panda et al., 2021) and would likely also decrease StHD severity in rice. GSH molecules protect cells by reducing accumulation of ROS (Larrainzar et al., 2014), and an increase in S was found to reduce Cd-induced toxicity in Brassica campestris (Anjum et al., 2008). It appears, then, that increasing S and GSH within a plant could reduce StHD severity in two ways, by increasing As-chelation and by increasing ROS scavenging. Other elements with known roles in induction of ROS scavenging include Si (Kim et al., 2017), Ca (Fichman and Mittler, 2021), and Cu (Ma et al., 2015).

The aim of the present study was to identify QTL and candidate genes that can enhance breeding efforts to limit the accumulation of As in rice grain (grain-As) and/or reduce the susceptibility of rice plants to As-induced stress, as reflected in StHD severity. Because genes that reduce the uptake or transport of As, as well as those that increase chelation and sequestration of As in vegetative tissues, could reduce both grain-As and StHD severity, our working hypothesis was that some QTL would be associated with reductions in both grain-As and StHD. Co-location with Si, P, S, Ca, or Cu QTL would direct candidate gene identification by revealing if the underlying gene altered upward As transport, sequestration, or ROS. While QTL for these individual traits have been identified in various populations, the present analysis of the traits together in one population improved the ability to evaluate trait-to-trait relationships and more precisely evaluate co-location among QTL to provide insight on underlying gene functions. The severity of the StHD disorder was used as a measure of susceptibility to As-induced stress. The concentration of Si in rice hulls (hull-Si) reflected differences in Si uptake and upward transport to panicles and grains, and grain concentrations of As, P, S, Ca, and Cu were used as a measure of element availability and upward transport.

## 2 Materials and Methods

### 2.1 Genetic Materials and Genotypic Data

Trait-to-trait relationships and QTL were identified using phenotypic and genotypic data on the USDA-ARS Rice Minicore Collection (RMC), a set of 202 pure line rice (Oryza sativa) accessions collected from 14 global rice-growing regions (Agrama et al., 2009). The RMC was previously used for GWAS using a marker map consisting of 156 PCR-based molecular markers distributed across the twelve rice chromosomes, including identification of hull-Si QTL (Bryant et al., 2011). Subsequent to that study, 173 RMC accessions were resequenced (Wang et al., 2016), and a marker map containing 3,200,320 (3.2 million) filtered, reliable SNPs was created and used for GWA mapping (Huggins et al., 2019). The present study accumulated phenotypic data on the subset of 167 RMC accessions having a high-density marker map listed in Supplementary Table S1 which includes accessions from six genetic subpopulations in rice, specifically 30 accessions classified as being from the aus (AUS) subpopulation, 54 being indica (IND), 29 temperate japonica (TEJ), 28 tropical japonica (TRJ), and 6 aromatic (ARO), with the remaining 20 accessions being admixtures of two or more subpopulation groups. With the AUS, IND, and AUS/IND admixtures being considered members of the indica subspecies (INDAUS), there were 89 INDAUS accessions. Similarly, the study set includes 66 accessions of the japonica subspecies, identified as TEJ, TRJ, or TEJ/TRJ admixtures.

### 2.2 Rice Minicore Data on Silica, Arsenic, Phosphorus, Sulfur, Calcium, and Copper From Prior Studies

Bryant et al. (2011) evaluated the hull silica concentrations of RMC accessions using rice harvested from 2 replications × 2 locations (Stuttgart, AR, and Beaumont, TX) then used least square means (LSMeans) along with a low-density marker map (164 DNA markers) to identify 12 putative hull-Si QTL. Raw sample data were obtained and reanalyzed for the present study. As a subset of the USDA Rice Core Collection, the RMC was evaluated by Pinson et al. (2015) for grain concentrations of 16 elements, including As, P, S, Ca, and Cu, using grains harvested from 2 replications × 2 years × 1 location (Beaumont, TX) from both flooded and unflooded fields. For the present GWA analyses, raw sample data from flooded plots were obtained for grain concentrations (mg kg^−1^) of As, P, S, Ca, and Cu, hereafter called grain-As, grain-P, and so forth. Details of sample production, separation of hulls and grains, sample digestions, and laboratory equipment and methods for determining element concentrations are in Bryant et al. (2011) and Pinson et al. (2015).

### 2.3 *De Novo* Evaluation for Straighthead Disorder Resistance

While some of the RMC accessions had been included in previous StHD QTL mapping studies (Li et al., 2016; Li et al., 2017), numerous RMC accessions had not yet been characterized for StHD. The RMC was evaluated for resistance to MSMA-induced StHD in the USDA-ARS Straighthead Nursery at Stuttgart, AR over 2 years, using two replications planted on May 5, 2015, and four replications planted on May 30, 2016. Growing rice accessions with red- or purple-colored bran in the Stuttgart, AR Straighthead Nursery is prohibited to prevent pollen outcrossing into nearby seed production fields. We therefore evaluated StHD on the 156 brown bran RMC accessions using 2-row plots drill-seeded with 3 g seed per 1.5 m row to ensure a dense plant stand, with 0.3 m between rows and plots. Plots were arranged in a randomized complete block design, with plots of a StHD-susceptible (“Cocodrie,” PI 606331, Linscombe et al., 2000) and resistant (“Zhe733,” PI 629016) cultivar inserted approximately every 10 plots in order to assure consistent StHD symptoms throughout the study area. The same RMC were similarly planted concurrently in an adjacent field area not treated with “MSMA,” hereafter called Native soil plots. Monosodium methanearsonate (MSMA) was applied on the day of planting at a rate of 6.7 kg ha^−1^ using Target^®^ 6.6 (Luxembourg-Pamol, Inc., Houston, TX, United States) which corresponds with 1.6 kg As ha^−1^ and incorporated into the upper 15 cm of soil before planting. Fertilization of fields began a month prior to planting with incorporation of P (triple super phosphate, 20 kg P ha^−1^) and potassium (K) (muriate of potash, 56 kg K ha^−1^). Nitrogen fertilizer (112 kg N ha^−1^ as dry urea) was applied to the soil surface just before the permanent flood was established at the five-leaf stage. The flood was maintained throughout the growing season, until maturity of all plots, to ensure ideal conditions for StHD development. Weeds were controlled with 9.3 l ha^−1^ of propanil (3′,4′-dichloropropionanilide) mixed with 0.4 kg ha^−1^ of Quinclorac (3,7-dichloroquinoline-8-carboxylic acid; Facet^®^, BASF, Ludwigshafen, Germany) when the rice was at the four-leaf stage. The soil at the site is Dewitt silt loam (fine, smectitic, thermic, Typic Albaqualfs) (5% sand, 78% silt, 17% clay), and all fields in this study had been managed in a rice-soybean rotation for more than 20 years. With the Straighthead nursery area receiving a fresh application of MSMA prior to planting rice every other year, the total As (iAs + oAs) in the soil has increased over time to 13 ± 6 kg ha^−1^ (Maguffin et al., 2020) compared to 4 ± 2 kg As ha^−1^ in the Native soil field area (Norton et al., 2012b). Regardless of the increased soil As content, a fresh application of MSMA is required to ensure development of StHD disorder in the nursery plots.

The MSMA-treated and Native soil plots were rated for StHD at maturity, approximately 35–40 days after heading. Heading was defined as 50% of the plants per plot having at least 1 panicle at anthesis. Days to heading (DHD) is the number of days between planting and heading per plot, with DHDms denoting data from MSMA-treated plots, and DHDnt for data from Native soil plots. Straighthead severity was rated on a 0 to 9 scale based on a visual observation of floret sterility and panicle emergence from the flag leaf sheath, as described by Yan et al. (2005). The plant height (PHT) of three random plants per plot was measured also at maturity and averaged per plot. The PHT for this study was defined as the distance (cm) from the soil surface to the tip of the tallest leaf to accommodate the fact that panicles are often not fully exerted on MSMA-treated plants. As for DHD, PHTms denotes data from MSMA-treated plots, and PHTnt denotes data from the Native soil plots. The 2015 trial was planted using RMC accessions obtained from the USDA Genetic Stocks Oryza (GSOR) Collection (www.ars.usda.gov/GSOR); 2016 plantings used seed harvested from 2015 Native soil plots.

### 2.4 Statistical Analyses of Rice Minicore Collection Trait Data

Analyses of variance (ANOVAs) were conducted using the generalized linear mixed model (GLIMMIX) procedure in SAS version 9.4 (SAS Institute Inc., 2012). Least square means (LSmeans) were calculated using the GLIMMIX procedure, with replication considered as a random effect and the RMC accessions as fixed effects. For calculating LSmeans combined across environments (either locations or years), the statistical model considered environment and replications nested within the environment as random effects.

Trait distributions, summary statistics, and mean comparisons across the seven population panels were calculated in JMP 14 (SAS Institute Inc. 2018) using trait LSmeans. Best linear unbiased predictions (BLUPs) computed per trait from raw replication data were used for the Pearson correlations and Bayesian network analyses. After a review of the data distributions, it was decided to transform the grain element data to reduce the strong skewing of these trait datasets prior to GWA analyses. Raw element concentration data were log-transformed and new BLUPs calculated for GWAS.

### 2.5 Bayesian Network Analysis

Bayesian network learning is a multivariate probabilistic modeling which computes, through an iterative learning process, the relationship between random variables represented by “nodes” and probabilistic dependencies represented by “arrows” in a directed acyclic graph (DAG) (Pearl, 1988; Scutari, 2010). The DAG was computed using the R package “bnlearn” (Scutari, 2010), as described in more detail in Sharma et al. (2021). The Bayesian network (BN) model was trained with 10-fold cross validation to compute the DAG (Scutari, 2010). Each validation used a random 17 accessions as the validation set (VS = 17) with the rest of the RMC accessions serving as the training set (TS = 150). Trait BLUPs were computed anew using the lme4 R package (Bates et al., 2011), this time accounting for population structure by using a mixed-effect model with a simple nested family structure (Piepho and Williams, 2006) that considered subgroups in the RMC as blocks. The growth stage progression of the traits in the order of flowering (DHDnt), plant height (PHTnt), grain element compositions, and lastly StHD disease ratings (StHDms) was encoded in the BN model using blacklisted arcs to compute the DAG (Scutari 2010).

The “bnlearn” package learns by iteratively selecting and estimating the model. The procedure first uses feature selection to find “parents” and “children” relationships among traits in the Markov blanket by the Semi-Interleaved HITON PC algorithm. In our model, the dependence was analyzed using Student’s t-test for Pearson’s correlations with alpha at 0.1. The large values of alpha were used because it allows Markov blankets to initially involve traits that are weakly associated, so that they are not initially discarded. Next, the procedure uses structure learning to find the DAG by computing the conditional independence present in data with a score-based algorithm using a heuristic optimization technique. In each iteration, the candidate DAG is assigned a network score reflecting its goodness of fit, which the algorithm then attempts to maximize. Then across all iterations, the analysis selects the DAG that maximizes the Bayesian information criterion.

### 2.6 Rice Minicore Genome Wide Association Panels and Parameters

GWA was conducted using 7 different “panels” derived from the RMC data, including the full RMC population (“All”); four subpopulations AUS, IND, TEJ, and TRJ; and the two subspecies indica (INDAUS = IND + AUS + IND/AUS admixtures) and japonica (JAP = TEJ + TRJ + TEJ/TRJ admixtures). For each filtered panel, Tassel V.5 (Bradbury et al., 2007) was used to generate a centered identity by state (IBS) kinship matrix and to conduct principal component analysis (PCA). The number of principal components (PCs) included in the mixed linear model analysis of each panel was adjusted in the following manner to account for population structure within each panel. For GWAS of the full RMC, the first three PCs were used as covariates in the mixed linear model; two PCs were used with the INDAUS subspecies; 1 PC with the JAP subspecies and the IND subpopulation; and no PCs used for TEJ, TRJ, and AUS. Mixed linear models were performed in Tassel version 5 (Zhang et al., 2010) with the variance components estimated for each marker and no compression options.

### 2.7 Interpretation of Genome Wide Association Mapping Results

As described in Huggins et al. (2019), a script was used that identified associated chromosome regions from individual SNPs or groups of physically linked SNPs. Chromosome regions included 50 kb in both directions around each individual significant SNP and were extended to include nearby significant SNPs occurring within 200 kb. A “Peak SNP” was designated for each region, which corresponded to the SNP with the most significant *p*-value. The observed frequencies of the alleles present at the peak SNP as well as the allele effect value were outputted (Huggins et al., 2019). Quantile–quantile (QQ) and Manhattan plots were created using the R package qqman (Turner, 2014). SNPs with −log10(*p*) > 5 were considered significant, and increased stringency was applied during data interpretation as needed, such as when only one SNP in a region met the log10(*p*) = 5 threshold, or regions where all associated SNPs had rare alleles (in <6 accessions), in which cases QTL were not claimed in these results. Running GWA on multiple panels provides more opportunity to identify chromosomal regions containing QTL but also prevents identification of a single “most strongly associated” SNP per QTL since it is common for SNPs to not be polymorphic in all panels, causing panels to identify QTL *via* association with different SNPs. Following the long-held precedent of claiming the fewest number of genes fitting a genetic model, we declared a single QTL region rather than multiple QTL per trait when different panels identified different but closely linked SNPs, merging linked SNPs into a single QTL (or not) per the following criteria. When considering SNP peaks across panels, a single QTL was declared if the string of SNPs significantly associated with a trait in one or more panels and/or study environments did not have a gap >800 kb and the additive effects were consistent across the SNPs and panels (example, qStHD1-1 in Supplementary Table S2). A chromosomal region containing multiple associated SNPs was declared as two QTL when a gap >800 kb existed between associated SNPs (e.g., qStHD1-1 to qStHD1-2), or if there were no SNP gaps >800 kb, but the additive effect of the predominant allele changed from positive to negative within a string of associated SNPs (e.g., qSi10-2 to qSi10-3). QTL for different traits were considered to overlap if the SNPs significantly associated with the two traits were interspersed with each other or if the ends of the QTL regions were ≤500 kb apart.

### 2.8 Confirmation of Targeted SNPs Using Pivot Tables

The multiple SNPs identified by GWA analyses for each QTL region were further evaluated using pivot tables in Microsoft Excel to characterize their allele effects on additional traits, e.g., to evaluate the effect of Si QTL on StHD or grain-As. Most of the QTL identified in a subpopulation panel were also significant in the GWA of all RMC, allowing Table 2, discussion text, and the pivot analysis to focus on SNP peaks as identified in the GWAS of “All” RMC. Calcium was unusual among the traits with several QTL significant in one subpopulation and not also significant in the related subspecies or “All” panels.

### 2.9 Candidate Gene Identification

Annotated functions of genes within the QTL regions were evaluated to identify candidate genes underlying the QTL for traits central to this study. Gene annotations from multiple public databases were merged into one file for this effort (Huggins et al., 2019) and included the candidate genes for general biotic and abiotic stress response genes identified in Cohen and Leach (2019), merged with all gene annotations in the Os-Nipponbare-Reference-IRGSP-1.0 assembly (Ouyang et al., 2006; Kawahara et al., 2013), the Rice Annotation Project (RAP1; http://rapdb.dna.affrc.go.jp/, accessed 26 Sept. 2019) (Sakai et al., 2013), and Oryzabase (OrzbaseGeneListEn_20190424010057; https://shigen.nig.ac.jp/rice/oryzabase/download/gene, accessed 26 Sept. 2019), and in Cohen and Leach (2019), which examined general biotic and abiotic stress response genes in rice.

## 3 Results

### 3.1 Rice Minicore Collection Trait Summaries

**FIGURE 1:**
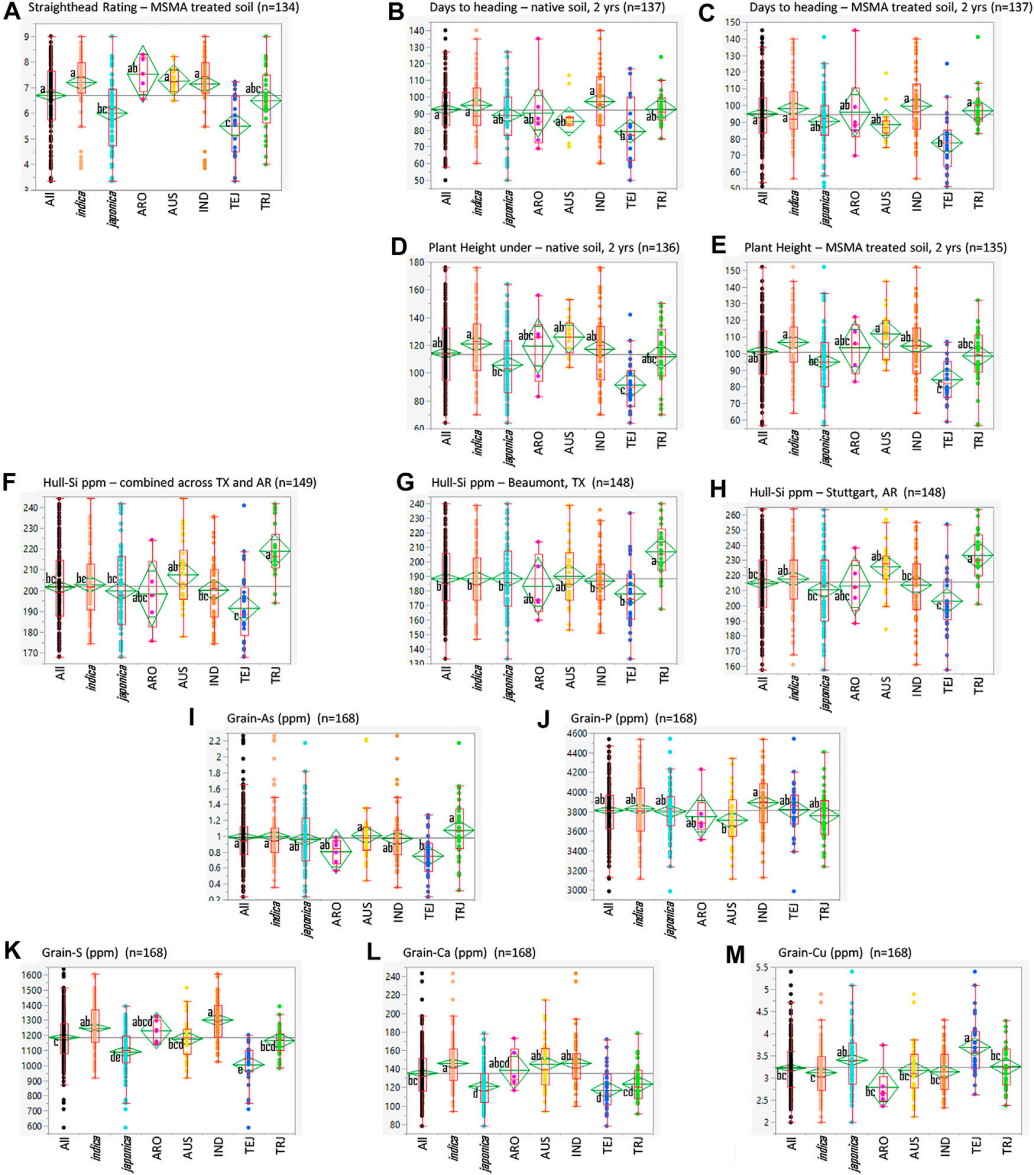
Quantile plots comparing the various traits in the entire USDA Rice Minicore (All) and the *O. sativa* subspecies (indica and japonica) and their subpopulations, aromatic (ARO), aus (AUS), indica (IND), temperate japonica (TEJ), and tropical japonica (TRJ). Lower and upper sides of boxes indicate the 25th and 75th percentiles, respectively; horizontal lines in boxes are medians, vertical lines indicate the 5th and 95th percentiles; and dots indicate full ranges of observed data. Difference among the small letters beside subpopulation means indicates differences among the means at *α* = 0.05.

The StHD ratings for the susceptible Cocodrie checks averaged 6.0 ± 0.9 in 2015 and 5.3 ± 0.7 in 2016 on the 0–9 rating scale (Yan et al., 2005). Zhe733 had notably less severe StHD symptoms with rating averages of 2.3 ± 0.5 in 2015, 2.1 ± 0.3 in 2016, and plot ratings ranging 2 to 3 in both years. Check plot ratings verified the production of MSMA-induced symptoms across the experimental areas in both years. Among the RMC, the average StHD 2-year LSmean in MSMA-treated plots was 6.7 ± 1.3 and ranged from 3.4 to 9 (Figure 1), with none of the accessions being as StHD resistant as Zhe733, the resistant check. The majority of the RMC (101 of 156 accessions) were as or more susceptible than Cocodrie, with LSmeans >6.0. None of the plots planted in the Native soil treatment received StHD ratings >3.

Summaries of all traits observed across the entire RMC (“All”) as well as divided among the subspecies (indica and japonica) and among the subpopulations (ARO, AUS, IND, TEJ, TRJ) are presented in Figure 1. Table 1 presents Pearson correlation coefficients between the trait BLUPs for the whole RMC (“All”) and for the indica and japonica subspecies. Subpopulation correlations are not presented or discussed further because their smaller panels identified relatively few significant correlations and their correlation patterns were similar to those of the indica and japonica subspecies panels. On average, the As-induced stress in the MSMA plots delayed DHD by 2 days and decreased height by 13 cm compared to the Native soil plots, but both DHD and PHT were highly correlated between the MSMA and Native soil treatments (*r* = 0.90 and 0.79, respectively, Table 1). While hull-Si was lower in Beaumont, TX (avg. 188 mg kg^−1^), compared to Stuttgart, AR (avg. 215 mg kg^−1^), the hull-Si calculated across the two locations was highly correlated with the Si data from each individual location (r = 0.86 for both Beaumont, TX, and Stuttgart, AR, Table 1). A positive correlation was observed between PHT and DHD under both Native and MSMA conditions among all panels in this RMC study.

**TABLE 1:** Pearson correlations between traits across all the Rice Minicore accessions, and across the indica and japonica subspecies panels.

All Rice Minicore Accessions (*n* = 167)	DHD native	DHD MSMA	PHT native	PHT MSMA	Si combined	Si Bmnt	Si Stgt	As	P	S	Ca	Cu
StHD	0.16	0.22*	0.39****	0.18*	−0.04	−0.02	−0.05	0.23*	0.17	−0.02	0.16	−0.31***
DHDnative	—	0.9****	0.28**	0.21*	−0.12	0.16	−0.34***	0.16	0.28**	0.06	0.13	−0.33***
DHDmsma	—	—	0.3***	0.17	−0.05	0.23*	−0.3***	0.29**	0.27**	0.12	0.07	−0.34***
PHTnative	—	—	—	0.79****	0.06	0.15	−0.02	0.36****	0.1	0.02	0.18*	−0.35***
PHTmsma	—	—	—	—	0.13	0.22*	0.03	0.3***	0.04	−0.06	0.19*	−0.32***
Si-Combined	—	—	—	—	—	0.86****	0.86****	0	−0.4****	0.03	0.24**	−0.05
Si-Bmnt	—	—	—	—	—	—	0.58****	0.16	−0.3***	−0.01	0.21*	−0.22*
Si-Stgt	—	—	—	—	—	—	—	−0.16	−0.38****	0.06	0.2*	0.1
As	—	—	—	—	—	—	—	—	0.11	0	0.14	−0.41****
P	—	—	—	—	—	—	—	—	—	0.02	−0.04	−0.07
S	—	—	—	—	—	—	—	—	—	—	0.05	−0.14
Ca	—	—	—	—	—	—	—	—	—	—	—	−0.28**
Cu	—	—	—	—	—	—	—	—	—	—	—	—
Indica accessions (*n* = 89)	DHD native	DHD MSMA	PHT native	PHT MSMA	Si combined	Si Bmnt	Si Stgt	As	P	S	Ca	Cu
StHD	0.45***	0.49****	0.52****	0.36**	−0.14	−0.03	−0.2	0.36**	0.33**	−0.13	0.16	−0.52****
DHDnative	—	0.93****	0.31*	0.17	−0.15	0.16	−0.4**	0.12	0.43***	0.04	0.02	−0.34**
DHDmsma	—		0.35**	0.13	−0.27*	0.08	−0.52****	0.19	0.47***	0.14	−0.04	−0.34**
PHTnative	—	—	—	0.74****	0.04	0.2	−0.13	0.41***	0.17	0.01	0.14	−0.47***
PHTmsma	—	—	—	—	0.25*	0.32*	0.1127	0.24	0.01	−0.14	0.15	−0.32*
Si-Combined	—	—	—	—	—	0.83****	0.80****	−0.04	−0.5****	−0.25	0.32**	−0.07
Si-Bmnt	—	—	—	—	—	—	0.33**	0.13	−0.33**	−0.13	0.31*	−0.29
Si-Stgt	—	—	—	—	—	—	—	−0.21	−0.5****	−0.29	0.21	0.18
As	—	—	—	—	—	—	—	—	0.14	−0.01	0.08	−0.42***
P	—	—	—	—	—	—	—	—	—	0.06	−0.04	−0.1
S	—	—	—	—	—	—	—	—	—	—	−0.19	0.03
Ca	—	—	—	—	—	—	—	—	—	—	—	−0.2
Cu	—	—	—	—	—	—	—	—	—	—	—	—
Japonica accessions (*n* = 66)	DHD native	DHD MSMA	PHT native	PHT MSMA	Si combined	Si Bmnt	Si Stgt	As	P	S	Ca	Cu
StHD	−0.29*	−0.13	0.18	−0.03	0.02	−0.04	0.06	0.10	−0.04	0	−0.02	−0.05
DHDnative	—	0.85****	0.23	0.27	−0.05	0.22	−0.28	0.3*	−0.09	−0.08	0.33*	−0.44**
DHDmsma	—	—	0.24	0.23	0.17	0.41**	−0.07	0.46***	−0.12	0.17	0.26	−0.49***
PHTnative	—	—	—	0.78****	0.11	0.11	0.11	0.31*	−0.03	−0.03	0.13	−0.28*
PHTmsma	—	—	—	—	0.04	0.15	−0.05	0.39**	0.01	−0.1	0.16	−0.36*
Si-Combined	—	—	—	—	—	0.89****	0.91****	0.02	−0.31*	0.29*	0.18	0
Si-Bmnt	—	—	—	—	—	—	0.62****	0.19	−0.28*	0.17	0.19	−0.16
Si-Stgt	—	—	—	—	—	—	—	−0.14	−0.27	0.34*	0.13	0.13
As	—	—	—	—	—	—	—	—	0.08	0.05	0.24	−0.47***
P	—	—	—	—	—	—	—	—	—	−0.17	−0.18	0.02
S	—	—	—	—	—	—	—	—	—	—	−0.05	−0.12
Ca	—	—	—	—	—	—	—	—	—	—	—	−0.24
Cu	—	—	—	—	—	—	—	—	—	—	—	—

Asterisks indicate significance at *α* = 0.05 *, *α* = 0.01 **, *α* = 0.001 ***, *α* = 0.0001 ****. All trait data were from rice grown in flooded field plots to maximize arsenic uptake and straighthead severity. Straighthead (StHD), days to heading (DHDmsma), and plant height (PHTmsma) were evaluated in 2015 and 2016 in Stuttgart, Arkansas, United States, using plots in MSMA-treated field area. DHDnative and PHTnative were evaluated the same year in the Stuttgart, Arkansas field area with “native soil” (not treated with MSMA); silica (Si) concentrations were evaluated using hulls from rice harvested 2 replications × 1 year from both Beaumont, Texas, and Stuttgart, Arkansas; concentrations of arsenic (As), phosphorus (P), sulfur (S), calcium (Ca), and copper (Cu), and -Cu were measured in grains harvested 2 replications × 2 years in Beaumont, TX.

Among the subpopulations, the TEJ were notably more resistant to StHD, earlier, and shorter (Figure 1). The TEJ were also lower in hull-Si, grain-As, grain-S, and grain-Ca, but higher in grain-Cu. This exemplifies the need to account for population structure when conducting GWA analyses across multiple subpopulations, accomplished in this study by using PCs as covariates.

### 3.2 Relationships Among Rice Minicore Collection Traits Indicated by Pearson Correlations and Bayesian Network

StHD and grain-As were always positively correlated, although the correlations were significant in the entire population (*r* = 0.23, *p* = 0.011) and among the indica accessions (*r* = 0.36, *p* = 0.003), but not significant among the japonica accessions (r = 0.50, *p* = 0.10) (Table 1). Contrary to our working hypothesis, Si, P, S, and Ca were not associated with either StHD or grain-As. Copper was negatively correlated with StHD, as predicted if Cu reduces StHD severity by increasing ROS scavenging, but Cu was also negatively correlated with grain-As. In fact, the strongest correlations with StHD are PHT from Native soil (PHTnt) (*r* = 0.39, *p* < 0.0001) and Cu (*r* = −0.31, *p* < 0.001), followed by grain-As (*r* = 0.23, *p* < 0.05), and the traits most strongly correlated with grain-As are Cu (*r* = −0.41, *p* < 0.0001) and PHTnt (*r* = 0.36, *p* < 0.0001), suggesting that correlations with PHT might be underlying the correlations observed between Cu with either StHD or grain-As.

**FIGURE 2:**
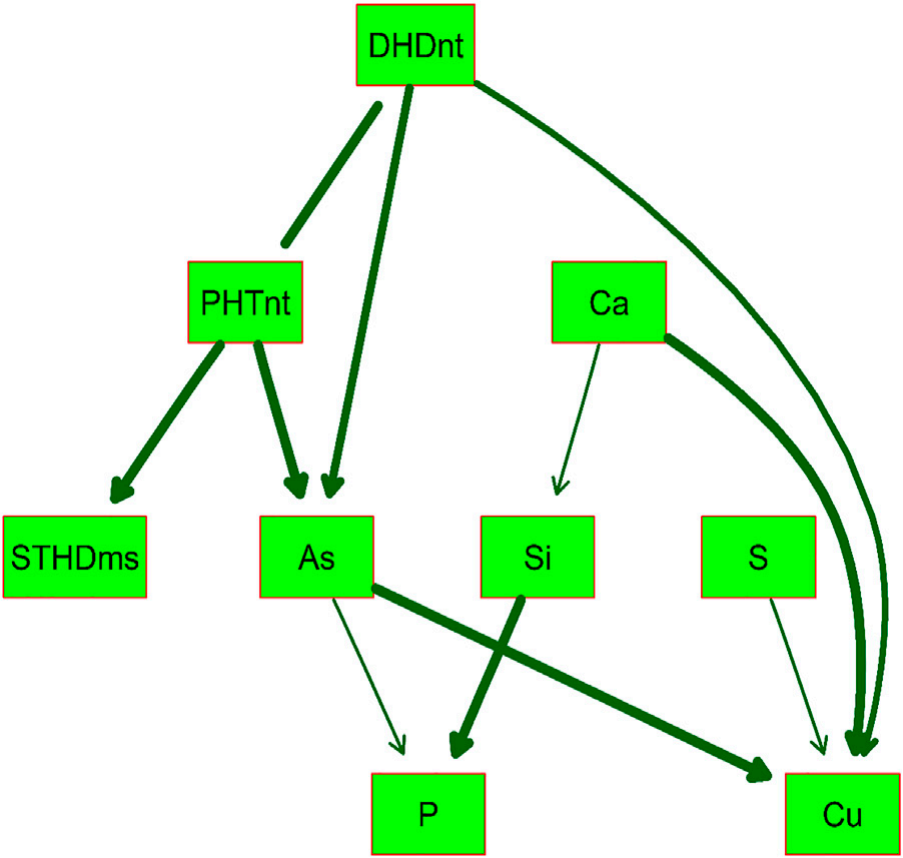
Directed acyclic graph (DAG) of the validated Bayesian network for nine arsenic-related traits determined in the USDA-ARS Rice Minicore (RMC) population with significance level of *p* = 0.001. The thickness of arrows between traits in green boxes represents the strength of the relationship. Straighthead disease severity was determined over 2 years using MSMA-treated soil (StHDms) in Stuttgart, AR. Days to heading (DHDnt) and plant height (PHTnt) were determined in the same 2 years and location using a native soil area (not treated with MSMA). Hull silica concentration (Si) was determined using rice grown in 1 year, two locations (Beaumont, TX and Stuttgart, AR), and two replications each. Grain concentrations of arsenic (As), phosphorus (P), sulfur (S), calcium (Ca), and copper (Cu) were determined in grains produced using 2 replications × 2 years at Beaumont, TX.

BN analysis is useful for predicting the likelihood that any one of several possible causes was the contributing factor to an observed event and presents the modeled predictions graphically using uni- or bidirectional arrows in the resulting DAG. Because of the high correlations (*r* > 0.75) observed between PHT and DHD in both Native and MSMA soils, and between hull-Si calculated across both locations with the hull-Si per individual location (*r* > 0.80), the BN analysis included only one version of these traits, namely, DHDnt, PHTnt, and hull-Si combined by BLUP across locations. Figure 2 presents the DAG determined by BN to best fit the phenotypic data from “All” RMC. The relationship between DHD and PHT was considered bidirectional (no arrow points on the connecting line), while all other relationships included in the BN model are unidirectional cause–effect relationships. The DAG indicates that the Pearson correlations observed between StHD, grain-As, and grain-Cu are likely caused by all three traits being affected by DHD, either directly or indirectly through PHT. The DAG also indicates no direct relationships between Si, S, Ca, and Cu with neither StHD nor grain-As. The DAG indicates a directional effect of As on P, a relationship which was positive but not significant in the Pearson correlations.

### 3.3 GWA-QTL Identified

**FIGURE 3:**
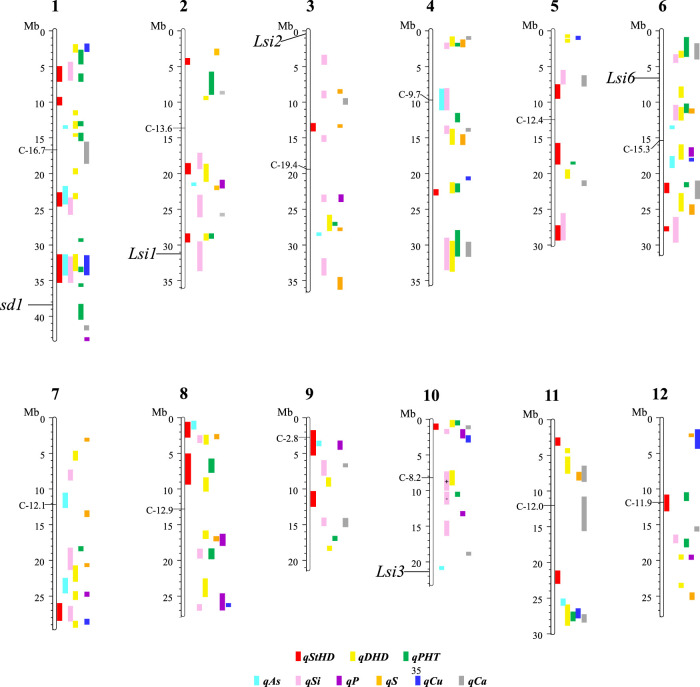
The physical position of the QTL for straighthead disease severity (qStHD), days to heading (qDHD), plant height (qPHT), hull silica concentration (qSi), and grain concentrations of arsenic (qAs), phosphorus (qP), sulfur (qS), calcium (qCa), and copper (qCu) identified by genome-wide association (GWA) mapping in the USDA Rice Minicore (RMC) with 3,200,320 SNP markers across the entire rice genome. Chromosome and megabase (Mb) positions of QTL and centromeres (C-Mb) are based on the Os-Nipponbare-Reference-IRGSP-1.0 assembly (Kawahara et al., 2013). The details of the QTL and their distinguishing SNPs are organized per trait with Si in Table 2, StHD in Table 3, As in Table 4, and the remaining traits in Supplementary Table S2.

Figure 3 presents the 195 GWA-QTL identified among the RMC for nine traits. Tables 2–4 present the QTL for Si (33 QTL), StHD (23), and As (15), respectively, while Supplementary Table S3 presents the QTL identified for the remaining As-related traits, P (11), S (18), Ca (19), Cu (9), DHD (39), and PHT (28). Manhattan and QQ plots are in Supplementary Figure S1. Because identification of a QTL in more than one population or environment not only validates that QTL but also demonstrates the reliability of the methods used to identify QTL in a particular study, when an RMC QTL was co-located with a previously reported QTL for the same or similar trait, this is noted in Tables 2–4 and Supplementary Table S3 by citing the previous QTL study or studies. Because the present study reevaluated the same data previously used to identify 12 hull-Si QTL (Bryant et al., 2011), we expected finding many of the same loci, and eight of the 33 Si RMC GWA-QTL did coincide with a QTL identified by Bryant et al. (2011) (Table 2). Seven additional Si RMC QTL coincided with QTL for hull, stem, or root Si concentrations reported by Dai et al. (2005) or Wu et al. (2006), and one encompassed the Lsi1 gene. These 16 validated Si QTL are now mapped more precisely in the present high-density GWA study. Of the four known Lsi transporter genes, only Lis1 on chr2 coincides with a RMC Si QTL (Figure 3; Table 2). Among the 23 StHD GWA-QTL (Table 3), seven were close (≤1.2 Mb distant) from a QTL previously reported for StHD (Agrama and Yan 2009; Pan et al., 2012; Li et al., 2016; Li et al., 2017), arsenite toxicity (Murugaiyan et al., 2019), or the chemical mimic, germanium toxicity (Talukdar et al., 2015), increasing confidence also in the 15 novel StHD RMC GWA-QTL. The most commonly identified StHD QTL was qStHD8-2, which co-located with QTL previously identified in four different populations (Pan et al., 2012; Li et al., 2016; Murugaiyan et al., 2019). When the RMC QTL for grain-As were compared with previously reported As QTL determined in various biparental mapping or GWA populations (Zhang et al., 2008; Norton et al., 2012a; Norton et al., 2014; Zhang et al., 2014; Norton et al., 2019; Liu et al., 2021), nine of the 15 RMC grain-As GWA-QTL coincided with a previously reported grain-As locus (Table 4). Four of the 11 RMC QTL for P, six of 18 for S, one of 19 for Ca, and 2 of 9 for Cu were validated by known transporter genes or previous QTL studies (Zhang et al., 2014; Liu et al., 2021) (Supplementary Table S3). Thirty-two percent (41/128) of the StHD and elemental QTL were validated by other studies, leaving 84 of the RMC QTL for As, Si, StHD, P, S, Ca, and Cu as novel. With the literature containing more than 200 reports of QTL affecting rice DHD and PHT, encompassing much of the rice genome, the present DHD and PHT GWA-QTL were compared only with genes confirmed to affect DHD or PHT. Seven of the 39 DHD QTL found among the RMC and 13 of the 28 PHT QTL encompassed a known DHD or PHT gene (Supplementary Table S3).

**TABLE 2:** QTL associated by GWA with hull-Si concentration (qSi) in the Rice Minicore Panel (RMC) arranged in chromosomal order.

QTL	Chr	Start of QTL region (bp)^a^	End of QTL region (bp)^a^	QTL size (Mb)	Peak SNP location (bp)^a^	Panel the QTL peak details are from^b^	-log10(p)	Effect of most common allele^c^	Most common allele	Alternate allele	Nu. acc. with common allele	Nu. acc. with alternate allele	% Panel having alt. All RMCele	QTL overlaps among traits in this RMC study	Co-location with QTL or genes for same/similar trait reported in literature	Candidate gene RAP ID^d^	Candidate gene symbol(s) or name(s)^e^
qSi1-1	1	44,82,438	70,15,096	2.533	69,65,096	All RMC	5.73	29.55	A	G	65	22	25.29	StHD, PHT	Dai et al. (2005), Bryant et al. (2011)	LOC_Os01g10600	NIP1;2
↓																LOC_Os01g10530	NIP1;5
↓																LOC_Os01g13120	TIP4;3
↓																LOC_Os01g13130	TIP4;2
qSi1-2	1	2,32,76,527	2,58,18,245	2.542	2,33,26,527	japonica	5.34	−25.32	G	A	35	6	14.63	StHD, As, DHD	Wu et al. (2006)	—	—
qSi1-3	1	3,17,24,740	3,51,81,598	3.457	3,18,15,255	All RMC	5.21	26.80	G	A	82	16	16.33	StHD, As, Cu, DHD	—	LOC_Os01g56050	MATE, multidrug and toxic compound extrusion
↓	1	3,17,24,740	3,51,81,598	3.457	3,44,82,225	All RMC	5.19	−21.87	T	C	104	11	9.57	—	—	—	—
qSi2-1	2	1,70,71,537	1,94,19,726	2.348	1,92,07,967	indica	6.34	−22.13	C	A	38	15	28.30	StHD	—	—	—
qSi2-2	2	2,30,94,460	2,61,30,899	3.036	2,31,44,460	All RMC	6.9	27.78	A	T	73	22	23.16	StHD, Ca, DHD	Bryant et al. (2011)	LOC_Os02g41860	PIP2;2
↓	2	2,30,94,460	2,61,30,899	3.036	2,52,54,352	indica	6.06	−19.80	A	G	30	24	44.44	—	—	LOC_Os02g44080	TIP2;1
qSi2-3 Bmt	2	2,96,10,452	3,39,69,000	4.359	2,99,81,669	indica	9.21	−42.71	T	C	51	8	13.56	DHD, PHT	Lsi1, Ma et al. (2008)	LOC_Os02g51110	Lsi1/NIP2;1
↓	2	2,96,10,452	3,39,69,000	4.359	3,33,14,549	japonica	5.06	−48.01	G	A	20	17	45.95	DHD, PHT	—	—	—
qSi3-1	3	34,09,465	48,02,256	1.393	43,20,769	All RMC	6.93	−29.80	C	T	67	25	27.17	none	Bryant et al. (2011)	LOC_Os03g08900	MATE, multidrug and toxic compound extrusion
↓					42,11,437	japonica	5.54	37.97	G	T	25	22	46.81	—	—	LOC_Os03g05390	SIET4
↓																LOC_Os03g05290	TIP1;1
qSi3-2	3	86,22,496	93,64,210	0.742	92,08,990	indica	5.43	−20.50	C	T	52	15	22.39	Ca, S	Wu et al. (2006)	—	—
qSi3-3	3	1,47,84,049	1,54,85,266	0.701	1,50,95,805	All RMC	5.78	36.00	G	A	77	30	28.04	none	—	—	—
qSi3-4	3	2,30,97,011	2,40,78,246	0.981	2,40,10,585	All RMC	6.11	−33.13	G	A	99	26	20.80	P	—	LOC_Os03g42830	MATE, multidrug and toxic compound extrusion
qSi3-5	3	3,20,21,749	3,43,04,026	2.282	3,20,71,749	All RMC	5.15	22.65	A	T	69	37	34.91	S	—	LOC_Os03g62270	MATE, multidrug and toxic compound extrusion
qSi4-1	4	16,89,594	25,49,585	0.860	23,60,309	All RMC	6.29	−33.76	G	A	83	5	5.68	S, Ca, DHD, PHT	—	—	—
qSi4-2	4	80,13,958	1,09,36,112	2.922	96,19,522	japonica	5.79	−44.70	A	T	22	18	45.00	As	Bryant et al. (2011)	LOC_Os04g16450	PIP2;6
qSi4-3	4	1,32,35,513	1,42,67,135	1.032	1,40,98,476	All RMC	7.54	−34.53	G	A	63	40	38.83	S, Ca, DHD	—	—	—
qSi4-4	4	2,90,64,644	3,36,00,846	4.536	3,09,35,902	All RMC	5.68	−18.30	C	T	53	37	41.11	Ca, DHD, PHT	—	LOC_Os04g47220	PIP1;2
↓																LOC_Os04g48290	MATE, multidrug and toxic compound extrusion
qSi5-1	5	53,95,155	75,34,826	2.140	54,74,098	japonica	5.29	44.40	T	C	22	19	46.34	Ca	—	LOC_Os05g11560	NIP1;2
↓	—	—	—	—	70,07,359	All RMC	8.25	−24.20	A	T	58	48	45.28	Ca	—	—	—
qSi5-2	5	2,56,26,649	2,92,13,988	3.587	2,84,84,725	All RMC	5.31	21.78	C	T	95	14	12.84	StHD	Dai et al. (2005), Bryant et al. (2011)	—	—
qSi6-1	6	32,68,717	44,53,379	1.185	43,66,202	All RMC	5.48	−30.92	T	A	83	26	23.85	DHD, PHT	Dai et al. (2005), (hulls&stem)	—	—
qSi6-2	6	1,03,69,048	1,23,06,167	1.937	1,04,19,048	All RMC	5.15	−25.19	T	A	78	7	8.24	DHD, S, PHT	—	LOC_Os06g22960	TIP2;2
qSi6-3	6	2,60,39,225	2,98,33,004	3.794	2,97,83,004	japonica	5.1	40.20	T	G	20	15	42.86	StHD	Bryant et al. (2011)	LOC_Os06g49310	MATE, multidrug and toxic compound extrusion
qSi7-1	7	72,60,858	86,83,035	1.422	73,10,858	All RMC	5.12	24.00	G	A	47	27	36.49	none	Bryant et al. (2011)	—	—
qSi7-2	7	1,82,08,134	2,13,43,107	3.135	1,91,86,954	All RMC	6.44	−21.70	C	T	82	31	27.43	S, DHD	Wu et al. (2006)	LOC_Os07g31884 & LOC_Os07g33310	Two MATE, multidrug and toxic compound extrusion genes
qSi7-3	7	2,62,82,630	2,85,75,856	2.293	2,76,83,847	All RMC	5.29	−22.00	C	T	48	39	44.83	StHD, Cu, DHD	Wu et al. (2006), Bryant et al. (2011)	—	—
aSi8-1	8	26,27,419	36,36,845	1.009	34,19,964	All RMC	5.42	−26.40	A	G	82	23	21.90	StHD, S, DHD	StHD	LOC_Os08g05580	NIP3;4
↓																LOC_Os08g05590	NIP3;2
↓																LOC_Os08g05600	NIP3;3
qSi8-2	8	1,86,74,724	1,97,78,306	1.104	1,96,29,742	All RMC	5.22	−20.00	G	A	56	39	41.05	PHT	—	—	—
qSi8-3	8	2,61,17,130	2,69,49,678	0.833	2,61,89,320	All RMC	5.27	−38.15	T	A	76	17	18.28	Cu, P	—	LOC_Os08g43250	MATE, multidrug and toxic compound extrusion
qSi9-1	9	60,70,051	81,35,783	2.066	63,71,780	indica	5.71	−32.78	G	A	50	8	13.79	Ca	Bryant et al. (2011)	—	—
qSi9-2	9	1,40,58,491	1,49,96,286	0.938	1,44,25,797	All RMC	5.27	48.80	C	A	77	16	17.20	Ca	—	—	—
qSi10-1	10	23,00,354	56,68,423	3.368	25,75,602	All RMC	6.36	−33.30	C	T	50	22	30.56	StHD, P, Ca, Cu, DHD, PHT	Wu et al. (2006)	—	—
↓					47,27,312	All RMC	6.59	−31.22	G	A	82	7	7.87	StHD, P, Ca, Cu, DHD, PHT	—	—	—
qSi10-2	10	73,49,091	1,00,66,729	2.718	90,34,052	All RMC	5.08	40.40	G	A	51	24	32.00	DHD	Wu et al. (2006)	—	—
qSi10-3	10	99,00,070	1,20,00,965	1.710	1,07,73,399	All RMC	6.18	−32.86	G	A	77	16	17.20	PHT	—	LOC_Os10g20350, LOC_Os10g20390, LOC_Os10g20450, LOC_Os10g20470	Cluster of 4 MATE, multidrug and toxic compound extrusion genes
qSi10-4	10	1,46,66,946	1,66,26,855	1.960	1,60,72,761	All RMC	6.27	−41.00	C	T	64	21	24.71	none	—	LOC_Os10g31040	SIET5
qSi12	12	1,64,64,615	1,74,50,987	0.986	1,74,00,985	japonica	6.41	36.00	T	G	19	14	42.42	PHT	—	—	—

Overlaps with QTL for other traits within this study, and candidate genes are also noted. Panels for which QTL are listed include “all RMC” containing all RMC accessions, indica subspecies, japonica subspecies, IND subgroup, and AUS subgroup.

a *O. sativa* SNPs are identified by their physical location based on the Os-Nipponbare-Reference-IRGSP-1.0 assembly (Kawahara et al., 2013).

b The panels are defined as the complete Rice Minicore Diversity Panel (RMC, n = 166). The *O. sativa* subpopulation groups were tropical japonica (TRJ), temperate japonica (TEJ), aus (AUS), and indica (IND). The two *O. sativa* subspecies are indica, composed of IND and AUS combined, and japonica comprised TEJ and TRJ. QTL were often identified in GWA-mapping of more than one population (e.g., in INDAUS and AUS). Because alternate alleles became very rare in the smaller subpopulations, the table presents the results based on the entire RMC panel when the QTL was significant there.

c A negative allele effect reflects a reduction in the trait associated with the most common allele.

d RAP ID is the Rice Annotation Project identification locus identified for the candidate gene.

e Gene nomenclature followed the standardized nomenclature for rice genes used in Oryzabase (Yamazaki et al., 2010).

**TABLE 3:** QTL associated by GWA with straighthead disease response (qStHD) in the Rice Minicore Panel (RMC) arranged in chromosomal order. Overlaps with QTL for other traits within this study, and candidate genes are also noted. Panels for which QTL are listed include “all RMC” containing all RMC accessions, indica subspecies, japonica subspecies, IND subgroup, and AUS subgroup.

QTL	Chr	Start of QTL region (bp)^a^	End of QTL region (bp)^a^	QTL size (Mb)	Peak SNP location (bp)^a^	Panel the QTL peak details are from^b^	-log10(p)	Effect of most common allele^c^	Most common allele	Alternate allele	Nu. acc. with common allele	Nu. acc. with alternate allele	% Panel having alt. All RMCele	QTL overlaps among traits in this RMC study	Co-location with QTL or genes for same/similar trait reported in literature	Candidate gene RAP ID^d^	Candidate gene symbol(s) or name(s)^e^
qStHD1-1	1	50,85,464	70,49,182	1.964	57,83,885	All RMC	5.18	1.48	T	C	61	17	21.79	Si, PHT	Agrama and Yan (2009)	LOC_Os01g10530	NIP1;5
↓																LOC_Os01g10600	NIP1;2
↓																LOC_Os01g11946	ABCD1
qStHD1-2	1	93,23,012	1,02,02,956	0.880	97,59,244	All RMC	5.52	2.26	C	T	60	15	20.00	none	Agrama and Yan (2009)	LOC_Os01g18670	ABCB1
qStHD1-3	1	2,26,92,755	2,46,17,484	1.272	2,33,95,696	All RMC	5.57	1.75	C	G	67	11	14.10	As, Si, DHD	—	LOC_Os01g41250 thru _Os01g41530	8 Fbox genes, Fbox021 thru Fbox028
↓																LOC_Os01g42430	vacuolar H + -ATPase subunit C
↓																LOC_Os01g42830	ABCI13
↓																LOC_Os01g42900	ABCG2
qStHD1-4	1	3,15,81,270	3,53,78,105	3.797	3,26,58,417	All RMC	5.88	1.98	C	T	60	12	16.67	As, Si, Cu, DHD, PHT	—	LOC_Os01g55210 thru _Os01g60920	11 Fbox genes, Fbox038 thru Fbox048
↓																LOC_Os01g56050	MATE, multidrug and toxic compound extrusion
↓																LOC_Os01g58290	Root development & fertility gene
qStHD2-1	2	37,23,340	48,46,184	1.123	37,73,340	All RMC	6.16	2.23	G	A	104	6	5.45	S	—	—	—
qStHD2-2	2	1,85,76,987	2,00,69,547	1.493	1,86,26,987	japonica	5.98	2.69	G	A	33	6	10.81	Si, DHD	—	—	—
↓					1,94,06,191	indica	5.09	2.63	G	C	30	6	11.76		—	—	—
qStHD2-3	2	2,87,47,996	2,94,74,023	0.726	2,92,36,156	indica	5.96	2.10	A	C	46	7	13.21	Si, DHD, PHT	—	LOC_Os02g45380	MATE, multidrug and toxic compound extrusion
qStHD3	3	1,30,69,711	1,40,35,564	0.966	1,31,19,711	All RMC	5.39	1.71	C	A	96	11	10.28	S	Pan et al. (2012)	—	—
qStHD4	4	2,21,73,581	2,23,30,955	0.157	2,22,23,581	All RMC	5.95	1.86	G	T	87	12	12.12	DHD, PHT	—	—	—
qStHD5-1	5	69,56,741	95,87,466	2.631	93,11,338	All RMC	5.03	2.55	C	T	85	5	5.56	Si, Ca	—	—	—
qStHD5-2	5	1,58,46,555	1,87,68,896	2.922	1,79,96,989	All RMC	5.43	1.53	A	T	83	15	15.31	PHT	Talukdar et al. (2015)	—	—
qStHD5-3	5	2,72,13,787	2,92,55,905	2.042	2,92,05,905	All RMC	5.78	1.66	T	A	52	42	44.68	Si	—	LOC_Os05g33910	MATE2, multidrug and toxic compound extrusion protein 2
qStHD6-1	6	2,12,68,226	2,27,22,667	1.454	2,13,32,716	All RMC	5.73	1.83	A	T	96	10	9.43	Ca, PHT	—	LOC_Os06g35930	NIP1;4
↓																LOC_Os06g36330	MATE, multidrug and toxic compound extrusion
qStHD6-2	6	2,78,50,453	2,80,83,810	0.233	2,79,26,998	All RMC	5.02	1.94	A	T	80	16	16.67	Si	—	—	—
qStHD7	7	2,60,79,985	2,84,51,701	1.935	2,76,86,009	All RMC	7.8	1.43	A	G	72	34	32.08	Si, DHD	—	—	—
qStHD8-1	8	4,50,806	28,59,799	2.409	28,09,799	All RMC	6.63	2.16	G	T	87	7	7.45	As, Si, S, DHD	—	LOC_Os08g03020	RLK1, membrane-anchored receptor-like kinase1
↓																LOC_Os08g03380	heat shock protein
↓																LOC_Os08g03470, 03480, 03490, 03500, 03510, 03530, 03650	7 BTB-domain containing genes: MB17, MB18, MBTB16 thruough MBTN19, BTBN17
↓																LOC_Os08g05580	NIP3;4
↓																LOC_Os08g05590	NIP3;2
↓																LOC_Os08g05600	NIP3;3
qStHD8-2	8	50,81,786	94,26,592	4.345	60,13,173	All RMC	6.59	1.49	C	T	59	19	24.36	DHD, PHT	Pan et al. (2012), Li et al. (2016), Murugaiyan et al. (2019)	LOC_Os08g10480	ATX1; antioxidant protein1
↓																LOC_Os08g09860	GLO6;glycolate oxidase6
↓																LOC_Os08g15149	Oxidoreductase
↓																LOC_Os08g15204	thioredoxin domain-containing protein 9
↓																LOC_Os08g15230	heat shock protein
↓																LOC_Os08g15330	anthocyanidin 3-O-glucosyltransferase
qStHD9-1	9	15,20,138	51,86,402	3.666	29,93,757	All RMC	6.35	2.88	A	G	41	34	45.33	As, P	—	LOC_Os09g03939	ABCG19
↓																LOC_Os09g06499	SULTR4;1, sulphate transporter4;1
↓																LOC_Os09g07450	flavonol synthase
↓																LOC_Os09g07670	ABC20
↓																LOC_Os08g08920	GLP8-1
↓																LOC_Os08g08970	GLP8-3
↓																LOC_Os08g08990	GLP8-5
↓																LOC_Os08g09000	GLP8-6
↓																LOC_Os09g09930	heavy metal transport/detoxification protein
qStHD9-2	9	1,01,82,415	1,23,66,105	2.184	1,22,98,325	All RMC	5.06	-2.38	A	C	59	23	28.05	none	Agrama and Yan (2009)	LOC_Os09g18390	flavonol synthase
↓	9															LOC_Os09g18450	flavonol synthase
↓	9															LOC_Os09g18470	Oxidoreductase
↓	9															LOC_Os09g18520	Oxidoreductase
↓	9															LOC_Os09g19650	ABCA6
↓	9															LOC_Os09g20000	heavy metal-associated domain containing protein
↓	9															LOC_Os09g20220	GST5, glutathione S transferase5
qStHD10	10	4,19,002	12,79,577	0.861	11,41,117	All RMC	7.24	2.13	G	A	75	8	9.64	Si, Ca, DHD, PHT		LOC_Os10g02300	PCR1, plant cadmium resistance1
↓																LOC_Os10g02350	transmembrane 9 superfamily member
↓																LOC_Os10g02750	PAP3B;purple acid phosphatase3B
qStHD11-1	11	26,20,435	36,39,030	1.018	26,73,334	All RMC	5.57	2.64	A	G	86	6	6.52	none	—	LOC_Os11g05700	ABCC16/MRP16, multidrug resistance-associated protein16
↓																LOC_Os11g05410	PAP20A;purple acid phosphatase20A
qStHD11-2	11	2,11,29,594	2,29,93,035	1.863	2,29,43,035	All RMC	5.5	2.04	G	A	92	6	5.15	none	Pan et al. (2012)	LOC_Os11g36430	AIR2, arsenic induced ring protein2
qStHD12	12	1,09,38,230	1,31,11,966	2.174	1,21,15,679	indica	6.86	3.05	G	A	15	10	40.00	PHT		LOC_Os12g22110	ABCG29
↓																LOC_Os12g22284	ABCG30

a *O. sativa* SNPs are identified by their physical location based on the Os-Nipponbare-Reference-IRGSP-1.0 assembly (Kawahara et al., 2013).

b The panels are defined as the complete Rice Minicore Diversity Panel (RMC, *n* = 166). The *O. sativa* subpopulation groups were tropical japonica (TRJ), temperate japonica (TEJ), aus (AUS), and indica (IND). The two *O. sativa* subspecies are indica, composed of IND and AUS combined, and japonica comprised TEJ and TRJ. QTL were often identified in GWA-mapping of more than one population (e.g., in INDAUS and AUS). Because alternate alleles became very rare in the smaller subpopulations, the table presents the results based on the entire RMC panel when the QTL was significant there.

c A negative allele effect reflects a reduction in the trait associated with the most common allele.

d RAP ID is the Rice Annotation Project identification locus identified for the candidate gene.

e Gene nomenclature followed the standardized nomenclature for rice genes used in Oryzabase (Yamazaki et al., 2010).

**TABLE 4:** QTL associated by GWA with grain arsenic concentration (qAs) in the Rice Minicore Panel (RMC) arranged in chromosomal order. Overlaps with QTL for other traits within this study, and candidate genes are also noted. Panels for which QTL are listed include “all RMC” containing all RMC accessions, *indica* subspecies, *japonica* subspecies, IND subgroup, and AUS subgroup.

QTL	Chr	Start of QTL region (bp)^a^	End of QTL region (bp)^a^	QTL size (Mb)	Peak SNP location (bp)^a^	Panel QTL peak details from^b^	−log_10_(p)	Effect of most common allele^c^	Most common allele	Alter-nate allele	Nu. acc. with common allele	Nu. acc. with alt. allele	% panel having alt. allele	QTL overlaps among traits in this study	Co-location with QTL reported in literature	Candidate gene RAP ID^d^	Candidate gene symbol(s) or name(s)^e^
*qAs1-1*	1	1,34,61,779	1,36,17,444	0.156	1,35,33,400	all RMC	5.56	−0.26	C	T	60	46	43.4	DHD, PHT	–	–	–
*qAs1-2*	1	2,19,81,983	2,44,49,843	2.468	2,37,57,196	all RMC	5.17	−0.11	G	T	71	39	35.5	StHD, Si, DHD	Norton et al., 2014	LOC_Os01g41720	*EnS-10*
*qAs1-3*	1	3,14,52,607	3,42,20,812	2.768	3,16,77,916	all RMC	5.67	0.15	C	T	108	21	16.3	StHD, Si, Cu, DHD, PHT	–	LOC_Os01g56400	*ABCI7*
*qAs2*	2	2,15,23,027	2,17,30,651	0.208	2,15,75,358	all RMC	5.31	0.27	G	A	52	43	45.3	P, S	–	LOC_Os02g36570	*ABC1-2*
*qAs3*	3	2,82,21,674	2,84,16,354	0.195	2,83,66,354	all RMC	5.91	−0.34	A	G	65	54	45.4	–	Zhang et al., 2008; Liu et al., 2021	LOC_Os03g49440	*phosphatase*
*qAs4*	4	82,22,722	1,12,92,867	3.070	90,09,797	all RMC	5.75	0.21	A	T	85	9	9.6	Si	–	LOC_Os04g16450	*PIP2;6*
*↓*																LOC_Os04g17660	*HAC1;2*
*qAs6-1*	6	1,30,39,217	1,31,69,010	0.130	1,31,15,883	*indica*	6.09	−0.14	C	T	55	11	16.7	–	–	LOC_Os06g22960	*TIP2;2*
*qAs6-2*	6	1,76,19,751	1,92,18,461	1.599	1,90,23,908	all RMC	5.03	0.17	G	T	106	7	6.2	DHD, Cu	Zhang et al., 2008	LOC_Os06g29790	*PHO1*
*↓*																LOC_Os06g29844, LOC_Os06g29950, LOC_Os06g29994	*Cluster of 3 MATE genes*
*↓*																	
*↓*																	
*qAs7-1*	7	1,05,06,160	1,25,89,848	2.084	1,18,74,171	all RMC	6.13	0.18	G	A	89	11	11.0	S	Norton et al., 2014	─	─
*qAs7-2*	7	2,26,36,921	2,45,33,222	1.896	2,26,86,921	all RMC	5.42	−0.16	G	A	82	31	27.4	DHD, P	Norton et al., 2014	LOC_Os07g41310	Similar to Phytochelatin synthetase
*qAs8*	8	3,94,274	18,15,831	1.422	5,98,139	all RMC	6.24	−0.37	A	G	43	39	47.6	StHD	Norton et al., 2014; Liu et al., 2021	LOC_Os08g03020	transmembrane receptor protein
*↓*																LOC_Os08g03380	heat shock protein
*↓*																	
*↓*																LOC_Os08g03470 to LOC_03650	7 BTB-domain containing genes
*↓*																	
*qAs10*	10	2,09,14,520	2,11,45,930	0.231	2,10,12,106	AUS	5.24	−0.09	A	G	10	9	47.4	–	–	LOC_Os10g37920	*MATE*
*↓*																LOC_Os10g39980	*Lsi3*
*qAs11-1*	11	83,21,863	89,01,679	0.580	88,40,750	*indica*	6.23	0.20	C	T	42	7	14.3	S, Ca	–	–	–
*qAs11-2*	11	2,50,34,091	2,59,95,458	0.961	2,52,09,544	all RMC	5.35	0.17	G	A	116	9	7.2	DHD	Norton et al., 2014	–	–

a *O. sativa* SNPs are identified by their physical location based on the Os-Nipponbare-Reference-IRGSP-1.0 assembly (Kawahara et al., 2013).

b The panels are defined as the complete Rice Minicore Diversity Panel (RMC, n = 166). The *O. sativa* subpopulation groups were tropical japonica (TRJ), temperate japonica (TEJ), aus (AUS), and indica (IND). The two *O. sativa* subspecies are indica, composed of IND and AUS combined, and japonica comprised TEJ and TRJ. QTL were often identified in GWA-mapping of more than one population (e.g., in INDAUS and AUS). Because alternate alleles became very rare in the smaller subpopulations, the table presents the results based on the entire RMC panel when the QTL was significant there.

c A negative allele effect reflects a reduction in the trait associated with the most common allele.

d RAP ID is the Rice Annotation Project identification locus identified for the candidate gene.

e Gene nomenclature followed the standardized nomenclature for rice genes used in Oryzabase (Yamazaki et al., 2010).

### 3.4 Co-Location Among GWA-QTL for Different Arsenic-Related Traits

Among the 23 StHD QTL and 15 As QTL, four were co-located (Tables 3, 4; Figure 3). Eleven of the StHD QTL and four of the As QTL coincided with Si. Although less common, P, S, Cu, and Ca QTL were found co-located with some StHD and As QTL. In all four instances of coincidence between StHD and As QTL, a pivot table evaluation of allele effects showed that the allele that decreased StHD at qStHD1-3, qStHD1-4, and qStHD8-1 also decreased As and Si. Similarly, the qStHD9-1 allele that decreased StHD severity also decreased both As and P. Thus, we did find some QTL overlaps lending support to the hypothesis that decreased As-uptake, as evidenced by decreased grain-As, hull-Si, or grain-P concentrations, results in decreased StHD as well. Because the DAG (Figure 2) suggested that overlap of StHD and As QTL might instead be caused by mutual dependence on DHD, it must be noted that all three instances of coincidence between StHD, As, and Si QTL also coincide with DHD. The one instance of overlap between P, StHD, and As (at qStHD9-1) does appear to be independent of DHD and PHT, as well as all other traits in this study (Table 3; Figure 3).

In agreement with the DAG (Figure 2) showing a strong influence of DHD on StHD *via* PHT, 10 of the 23 StHD QTL co-located with a PHT QTL, 8 with DHD, for a total of 12 that coincide with DHD, PHT, or both (Figure 3; Table 3). The DAG did not indicate a close relationship between StHD and Si, nor between Si and DHD. Even so, co-location among StHD and Si QTL was high, with 12 of the 23 StHD loci overlapping or being within 1 Mb of a Si QTL. This suggests that Si concentrations may in fact affect StHD severity in a manner not detected in this data by BN. For example, Si might affect StHD but less strongly than DHD and PHT. Similarly, four As QTL (qAs2, qAs3, qAs7-1, and qAs8-1), and three StHD QTL (qStHD1-1, qStHD3, and qStHD8-1) were on or near S QTL (Tables 3, 4; Figure 3), suggesting that increased As-chelation and/or increased ROS scavenging by increased S might be affecting grain-As or StHD. Overlaps between StHD and Ca or Cu QTL were notably uncommon and always involved another trait such as DHD or Si (qStHD1-4 and qStHD7 with Cu QTL, and qStHD5-1, qStHD6-1, and qStHD10 with Ca QTL), indicating that increased ROS scavenging from increased Ca or Cu is not contributing significantly to the observed variance for StHD resistance.

There are four instances of co-location between As and P QTL (chr 2, 6, 7, and 9; Table 4 and Figure 3), with pivot table analyses showing the allele that increased As also increased P. The instance of overlap on chr9 was discussed above as independent from DHD, but the other three do coincide with a DHD QTL. While the DAG does not show a direct connection between DHD and P, it does show a DHD effect on P channeling through As (Figure 2), and DHD-P correlations were significant and positive among the entire RMC and indica subspecies, but the DHD-P correlations were near zero among japonica accessions (Table 1).

## 4 Discussion

Factors affecting Si concentrations are candidates for factors affecting As, which are in turn candidates for StHD. We will therefore discuss the traits in that order.

### 4.1 Candidate Genes for Si QTL

To explore candidate genes in the 33 Si RMC GWA-QTL (Table 2), we searched annotated genes to find those involved with transmembrane transport of silicon transport or metals and metalloids. The four known Low silicon transporters (Lsi1, Lsi2, Lsi3, and Lsi6) are of two types, with Lsi2 and Lsi3 being silicon efflux transporters (SIETs), and Lsi1 and Lsi6 being aquaporins of the Nodulin-26 like intrinsic protein (NIP) type. Only one of the four Lsi genes, specifically Lsi1, coincided with an RMC Si QTL (Table 2; Figure 3). NIPs are a subclass of membrane intrinsic transporters (MIP) which include also plasma membrane intrinsic proteins (PIPs) and tonoplast intrinsic proteins (TIPs). Overall, across the 33 Si RMC GWA-QTL regions (Table 2), we identified as candidate genes two SIETs, seven NIPs, three PIPs, five (TIPs), and 13 multidrug and toxic compound extrusion (MATE) genes. All these genes were located within 1 Mb of 17 of the 33 Si RMC GWA-QTL.

### 4.2 Candidate Genes for As QTL

To explore candidate genes in the 15 As RMC GWA-QTL (Table 4), we again looked in QTL regions for components of silicon and heavy metal uptake or transport and added to the search genes for signaling, and/or tolerance mechanisms (Abedi and Mojiri 2020) as well as chelation and detoxifying enzymes reported in the literature as affecting metalloid metabolism (Chen et al., 2017; Abedi and Mojiri, 2020). Overall, across the 15 As RMC GWA-QTL regions, we identified three NIPs, one SIET/Lsi3, four MATE genes, two ABC family proteins, one PIP, one TIP, one phosphate transporter, two transmembrane proteins (i.e., transmembrane receptor protein and EnS-10, endosperm-specific gene with metal ion transmembrane transporter activity), three metabolizing enzymes (phosphatase, arsenate reductase (HAC1;2), and one similar to phytochelatin synthetase), and one heat shock protein as noted in Table 4. These genes were located within 1 Mb of 10 of the 15 As RMC GWA-QTL.

The candidate gene identified by the overlapping qSi4-2/qAs4 QTL, PIP2;6, was initially reported to be involved in influx and efflux of boron transport (Mosa et al., 2016), but the heterologous expression of OsPIP2;6 in *Xenopus laevis* oocytes was also found to increase the uptake of arsenite (Mosa et al., 2012). Several NIP proteins have been shown to transport As, including the well-known Lsi1 and Lsi6 aquaporin transporters, NIP1;1 (Kamiya et al., 2009), and Arabidopsis NIP3;1 (Xu et al., 2015). The NIP3;2 protein (candidate for qSi8-1) has also been shown permeable to arsenite (Bienert et al., 2008; Ma et al., 2008). However, while this Si QTL overlapped with qStHD8-1, it did not overlap with the nearby qAs8. MATE genes play an important role in cellular detoxification processes. Using a transcriptomic study, Seth et al. (2020) identified nine MATE genes that were upregulated upon exposure to As. The constitutive expression of OsMATE2 in transgenic tobacco plants decreased root-to-shoot As transfer, and transgenic rice plants wherein RNAi was used to silence OsMATE2 produced grains with less grain-As than wild-type rice plants (Das et al., 2018). While MATE2 was not among the grain-As candidate genes, it is a candidate gene for qStHD5-3.

### 4.3 Candidate Genes for StHD QTL

Because StHD has been attributed to As toxicity (Tang et al., 2020a), to explore candidate genes underlying the 23 StHD RMC GWA-QTL (Table 4), we included in the search the same components of Si and heavy metal uptake, transport, signaling, and/or tolerance mechanisms, chelating and detoxifying enzymes included in the As candidate gene search, and added also stress-protective agents (e.g., redox homeostasis and antioxidant defense). Across the 23 StHD RMC GWA-QTL regions (Table 4), we identified 10 ABC family transporter proteins, six NIPs, two MATEs, one Si transporter, two membrane proteins, eight enzymes (i.e., vacuolar H + -ATPase subunit C antioxidant protein 1 (ATX1), glycolate oxidase 6 (GLO6), flavonol synthase, oxidoreductase, glutathione S transferase 5 (GST5)), two purple acid phosphatases (PAP3B, PAP20A), and one plant cadmium resistance (PCR1) gene within 1 Mb of 12 of the 23 StHD RMC GWA-QTL. Vacuolar ATPase is a proton pump protein whose role in acidification of the vacuolar compartment activates the uptake and release of ions and metabolites (Sze et al., 1992; Lüttge and Ratajczak, 1997). Furthermore, ATX1 is known to interact with heavy metal P1B-ATPases, suggesting its role in delivering Cu to heavy metal P1B-ATPases for Cu trafficking and distribution in order to maintain Cu homeostasis in rice (Zhang et al., 2018). RLK1 encodes a plasma membrane-localized protein that acts upstream of mitogen-activated protein kinase (MPK) cascades and positively regulates defense-related MPKs and WRKY transcription factors to respond differentially to external stimuli (Meng and Zhang, 2013; Macho and Zipfel, 2014). The ubiquitin-proteasome system (UPS) is one of major protein regulation pathways that enhance the adaptation and survival of plants under various environmental stresses such as toxic metalloid exposure as well as other environmental stresses (drought, salinity, and cold). Recent study showed active E3 ligase activity of OsAIR2 through *in vitro* ubiquitination assay and further examined that overexpression lines of OsAIR2 in Arabidopsis improved the seed germination and increased the root length under arsenate stress conditions, suggesting its role as a positive regulator of As stress tolerance (Hwang et al., 2017). Germin-like protein (GLP) is a plant glycoprotein associated with the plant cell wall, and its various proposed roles in plant development and defense are known (Dunwell et al., 2008; Caliskan, 2009).

### 4.4 Further Consideration of Candidate Genes in Genomic Regions Containing Overlapping As and StHD QTL

The region of Chr8 encompassing qAS8 and qStHD8-1 (Chr8 0.45-0.82 MB) contains a series of BTB-domain-containing proteins (MB17&18, MBTB16, 17, 18, 19; BTBN17) that are also of interest. A BTB domain-containing protein was reported to be involved in enhancing iron homeostasis in apple (Zhao et al., 2016). Recent study also showed that rice plants overexpressing a BTB domain protein (OsTAZ24) promoted plant resistance against various heavy metals (Shalmani et al., 2021), demonstrating the role of the BTB-domain-containing protein in heavy metal homeostasis, making these genes candidates for both the grain-As and StHD QTL. We therefore list this cluster of BTB-domain proteins as candidate genes for both qAs8 (Table 4) and qStHD8-1 (Table 3).

There were two RMC GWA-QTL regions that were associated with Si along with As and StHD (qAs1-2/qStHD1-3/qSi1-2, and qAs1-3/qStHD1-4/qSi1-3). Interestingly, these two regions, one on Chr1 at 23.34–24.45 Mb and the other one on Chr1 at 31.72–34.22 Mb, contain eight and 11 F-box domain-containing proteins, respectively. Recent study showed that 12 F-box domain-containing proteins were located in the As QTL regions from a japonica diversity panel of 228 accessions and from 95 advanced breeding lines with japonica genetic backgrounds phenotyped for As concentration in the flag leaf as well as in dehulled grain (Frouin et al., 2019). One of the F-box proteins identified as a candidate gene in our study, OsBOX028, was also identified by Frouin et al. (2019), suggesting that F-box proteins may have a role in affecting As tolerance. F-box proteins are known to define the specific substrates of the SCF complexes that are part of the ubiquitin/26S proteasome pathway for degrading unwanted or misfolded proteins (Zheng et al., 2002; Lechner et al., 2006). Thus, these F-box gene complexes are listed as candidate genes for qStHD1-3 and qStHD1-4 QTL (Table 3), but not for the coincident As and Si QTL (Tables 2, 4).

Another interesting aspect of the QTL overlaps was a region on chr9 containing QTL for StHD and As but not for Si. A QTL for P (qP9) was also located with qStHD9-1/qAs9. When we further examined the genes in this overlapping region, we found that more than 50% of genes in this region were retro/transposon proteins, forty retrotransposon proteins and five transposon proteins out of 99 total genes in this overlapping QTL region of qAs9, qStHF9-1, and qP9 (Chr. 9, 3,356,592–3,937,895). Transposons and retrotransposons play a role in post-transcription regulation through silencing of transposable elements (TEs), which can elicit gene variation and functional changes (Gao et al., 2012). TEs are also a source of small RNAs. A role for small RNAs in regulation of P starvation has been reported (Fang et al., 2009; Hsieh et al., 2009; Chiou and Lin 2011; O’Rourke et al., 2013).

### 4.5 Potential Impacts of Silica on Arsenic and StHD not Caused by Shared Silica/Arsenic Transport

Eleven of the 33 StHD QTL were co-located with Si QTL in the RMC, only three of which were also co-located with As QTL. This suggests a benefit from Si on StHD that is not directly associated with As and shared Si–As transporters. Numerous recent studies have confirmed Si beneficial effects on a variety of plant species growing under a wide range of environmental conditions. Si not only has a role in ameliorating biotic and abiotic stress but also can alleviate nutrient deficiency and toxicities (Pavlovic et al., 2021). Most of the studies regarding Si nutrient interactions were studies conducted on Si accumulators like rice. A general model for Si interactions has been proposed based on those studies for silicon-mediated response to mineral deficiency and toxicity (Ali et al., 2020). Under nutritional stress, uptake of Si increases through Lsi transporters (Yamaji and Ma, 2011) or aquaporins (Deshmukh et al., 2013). The increase in Si induces genes encoding the uptake and translocation of minerals in roots and facilitates the increase or decrease uptake of the corresponding nutrients such as N (Wu et al., 2017) and P (Owino-Gerroh and Bascho, 2005). In shoots, Si interacts with phytohormones and amino acids, and metabolites lower oxidative stress by further modulating the antioxidant enzymes (Liang et al., 2003; Li et al., 2012; Chalmardi et al., 2014) and simultaneously maintaining or even inducing higher photosynthetic efficiency resulting in plant growth and development (Detmann et al., 2012). On the other hand, for mineral or heavy metal toxicity, Si forms a complex with some heavy metals (Cd) or minerals (Zn, Fe, Cu) in the root cell wall, therefore reducing uptake and translocation to the shoots (Liu et al., 2013; Kopittke et al., 2017; Nozawa et al., 2018; Bosnic et al., 2019). In shoots, Si accumulation further mitigates metal toxicity by sequestration of minerals (Cu, Zn) and heavy metals (As, Cd) into the vacuoles of leaf cells (Keller et al., 2015; Kopittke et al., 2017; Nozawa et al., 2018). Overall, Si has the potential of enabling the plant to react adaptively against nutritional stress and promotes tolerance.

### 4.6 Conclusion: Implications for Future Research

The initial hypothesis was that both StHD and grain-As would be regulated strongly enough by the concentration of free (unbound, non-sequestered) As in the plant that they would share numerous QTL. We further hypothesized that overlap of the StHD and As QTL with Si, P, or S would provide further insight as to whether the shared StHD and As QTL were being driven by As-uptake or post-uptake metabolism. Because data existed to add Ca and Cu to the study, and because these elements have been shown to increase ROS scavenging, which could in turn reduce StHD severity, they were included as well. Correlations were positive between StHD, As, and Si, as would be expected if our first two hypotheses were true. However, finding a stronger StHD-Cu correlation than seen for StHD-As was unexpected. The BN DAG results cautioned to not overlook possible confounding effects from DHD in our data interpretations. When QTL were identified, and overlaps considered, the number (4 total) and percentage of StHD (0.12) and As (0.27) QTL that were co-located were lower than hypothesized. We also predicted that Si would impact StHD *via* its influence on As uptake. In contrast, we found that significantly more StHD QTL overlapped with Si QTL (11/33) than with As QTL (4/33). The results indicate that StHD and grain-As are both more complex than initially hypothesized and indicate that Si QTL may be affecting StHD response in ways not connected by As concentration, such as by increasing ROS scavenging, or improving overall plant health and nutrient balance.

Even though the hypothesis based on an StHD-As association was not well supported, the StHD and Si QTL identified among the RMC accessions in this study are the first to be identified using high-density mapping and, thus, are mapped to smaller QTL regions than previously available for these traits. Furthermore, the high proportion of QTL identified among the RMC accessions that were validated from those in literature indicates that the QTL-identification methods used were robust, increasing confidence in both the validated and novel QTL we report. The novel Si QTL, in particular, are worth deeper investigation since many of them were not explainable by currently known Si transporters and because Si did appear to have a stronger impact on StHD than could be explained by As alone. The region of chr8 that has been commonly reported as associated with StHD (qStHD8-2) also warrants further investigation. This qStHD8-2 QTL region was not associated with either As or Si but was found to contain multiple genes of interest including genes for antioxidants and redox factors which could be reducing ROS damage, suggesting that differences in ROS damage should be considered in future research on StHD resistance mechanisms. The region of chr9 containing qStHD9-1, qAs9, and qP9 which was independent from DHD would also be a genomic segment warranting further research. Other genes of interest for future study, because they were found in multiple grain-As and StHD regions, include several multidrug and toxic compound extrusion (MATE) genes, F-box genes, BTB-domain proteins, and NIPs not documented to date to transport As. The candidate genes we identified could be a basis for gene editing studies to determine the genes underlying the StHD, As, and Si QTL identified in the RMC.

## Data Availability

The trait data used for the present GWA analyses are in Supplementary Table S1. Genotypic datafiles developed by Huggins et al. (2019) for the Rice Minicore Collection can be downloaded in either VCF or HapMap format at https://www.ars.usda.gov/southeast-area/stuttgart-ar/dale-bumpers-national-rice-research-center/docs/mini-core-collection/.
