# Supplementary material for: Relationships Among Arsenic-Related Traits, Including Rice Grain Arsenic Concentration and Straighthead Resistance, as Revealed by Genome-Wide Association
Source: Front Genet. 2022 Mar 14;12:787767. doi: 10.3389/fgene.2021.787767 (PMC8974240; doi:10.3389/fgene.2021.787767)

Supplementary Figure S1: Manhattan (left) and Q-Q (right) plots identifying the genomic regions identified by GWA analyses as associated with one of the arsenic-related traits (Table 2). In the Manhattan plots the X axis shows the SNP positions across the 12 rice chromosomes and the Y axis is the –log10 (p) value for each SNP. The black horizontal line represents the –log10(p) threshold at 5. QTL numbers associated with the SNP peaks are indicated per trait. The plots are organized by trait, with the Straighthead disease results in plots A through C; hull silica results in plots in D through F; grain element concentrations of arsenic (As, G to I), phosphorous (P, in J), sulfur (S, in K), calcium (Ca, in L to O), copper (Cu, in P); heading date (DHD, in Q to S), and plant height (PHT, in T to V).

(A) Straighthead across 2 years – All Minicore

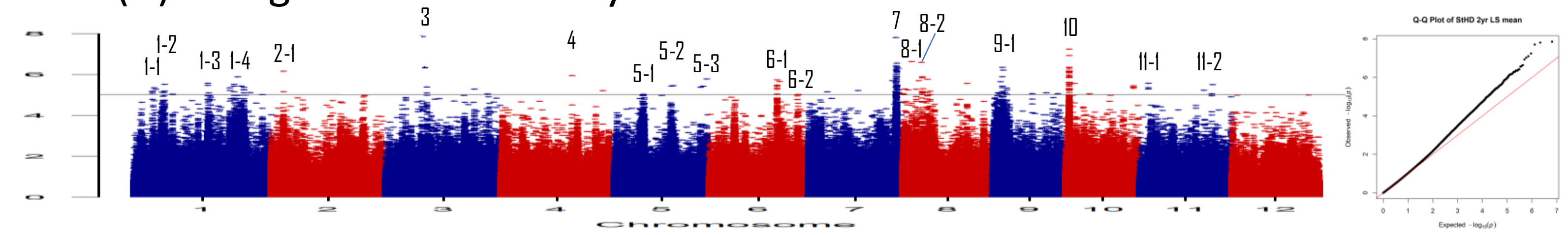

(B) Straighthead across 2 years – *indica* subspecies

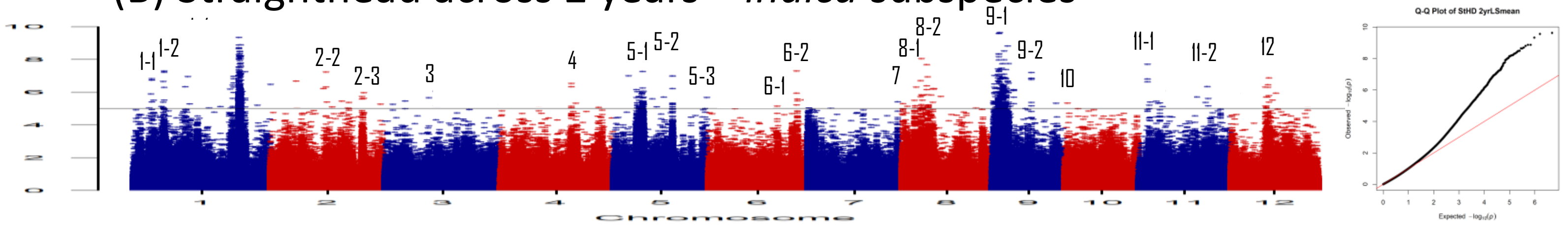

(C) Straighthead across 2 years – *japonica* subspecies

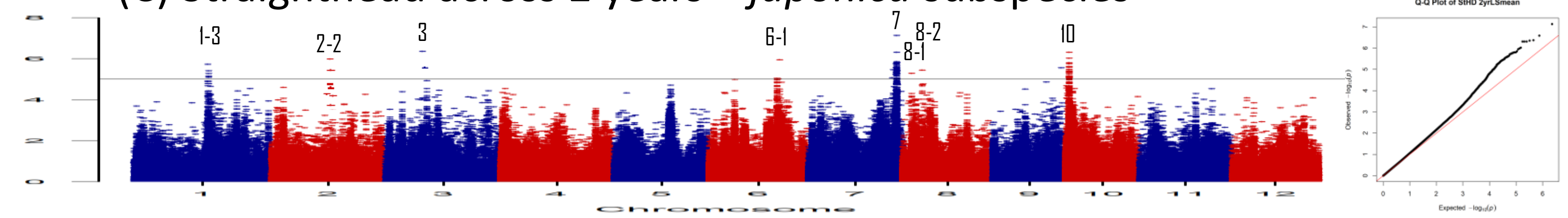

(D) Hull Si-concentration across 2 locations – All Minicore

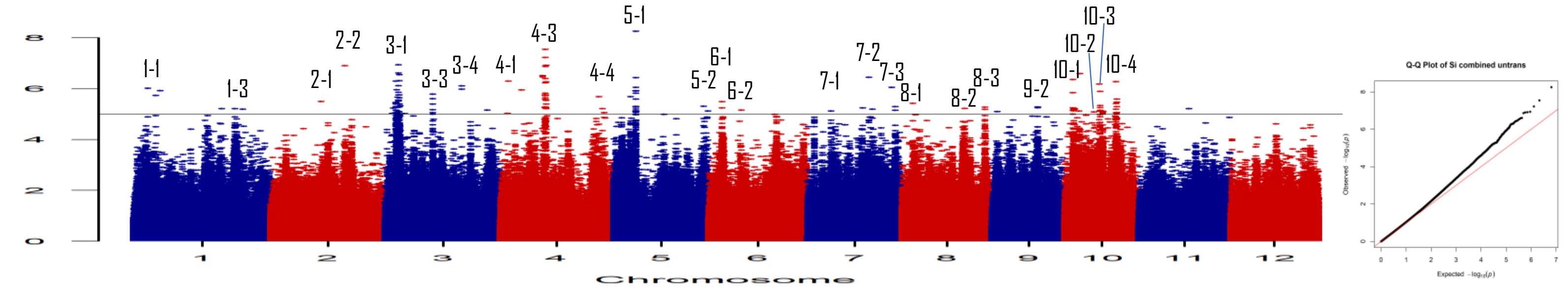

(E) Hull Si-concentration – analyzed across 2 locations, *indica* subspecies

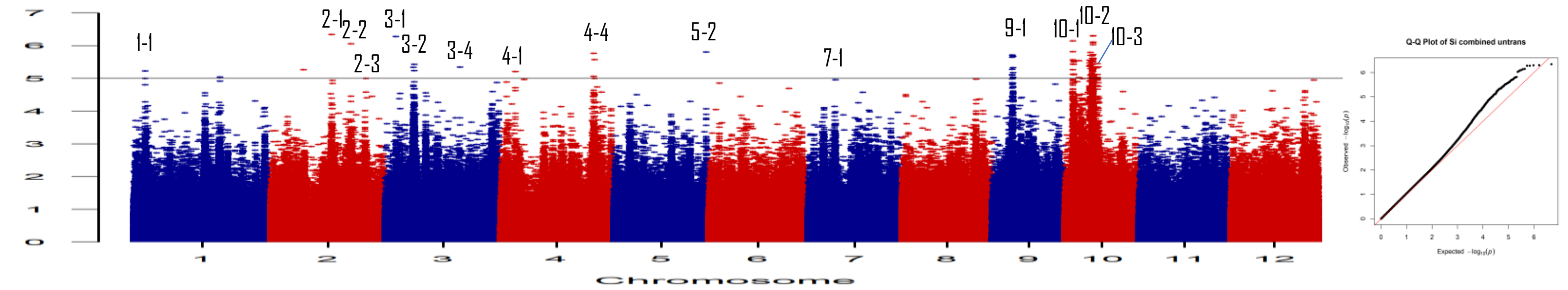

(F) Hull Si-concentration – analyzed across 2 locations, *japonica* subspecies

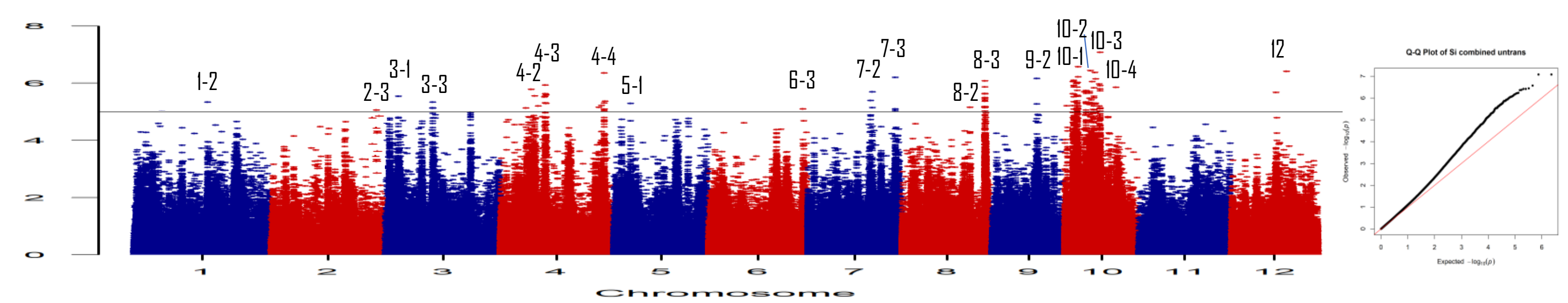

(G) Grain-Arsenic concentration across 2 years – All Minicore

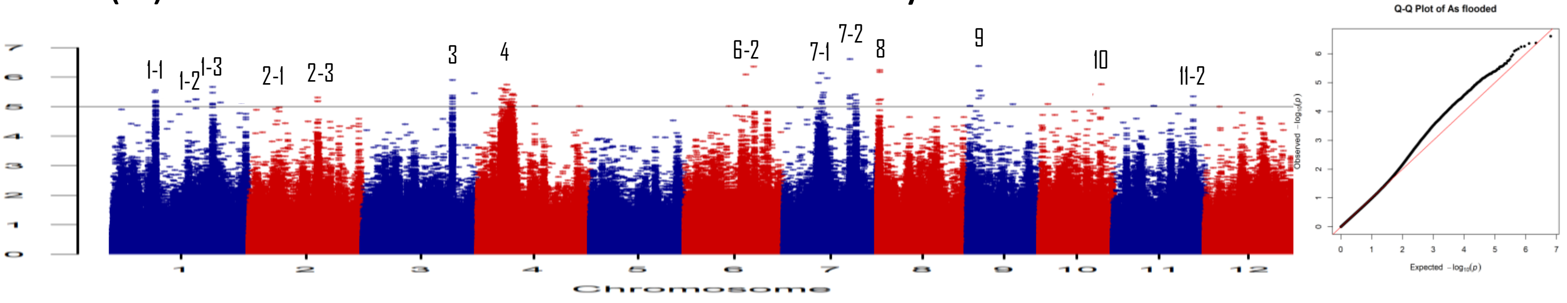

(H) Grain-As concentration – analyzed across 2 years, *indica* subspecies

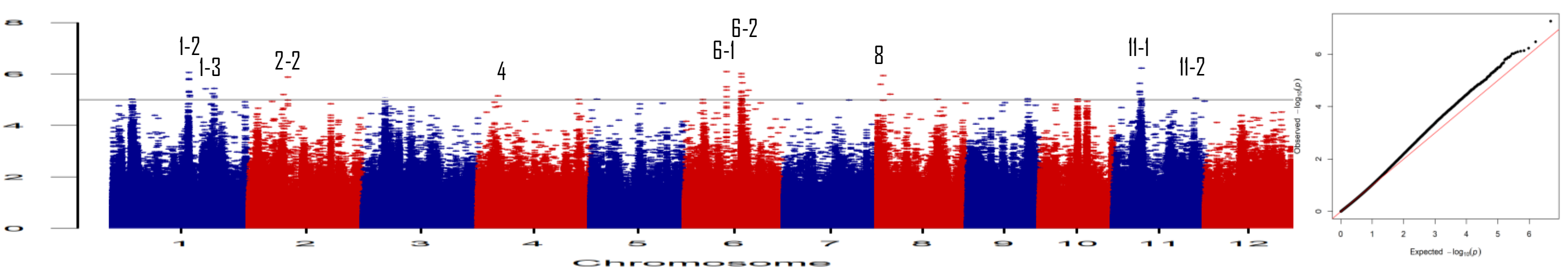

(I) Grain-As concentration – analyzed across 2 years, *japonica* subspecies

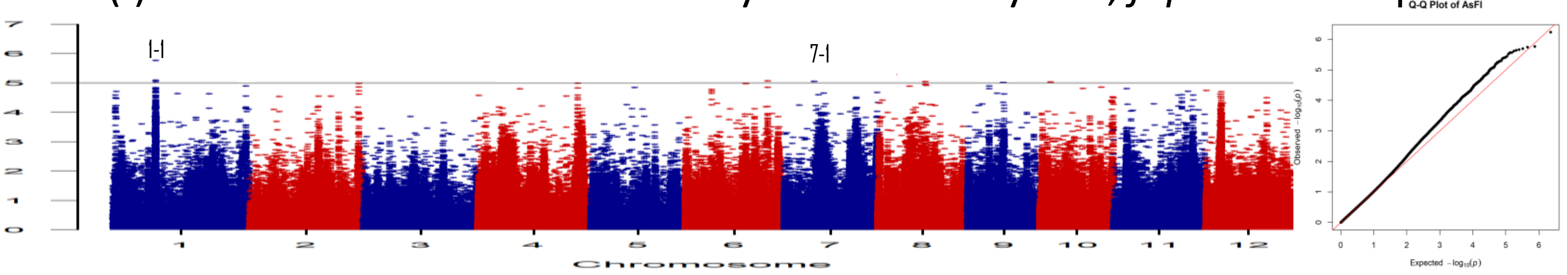

(J) Grain-P concentration – analyzed across 2 years, all Minicore

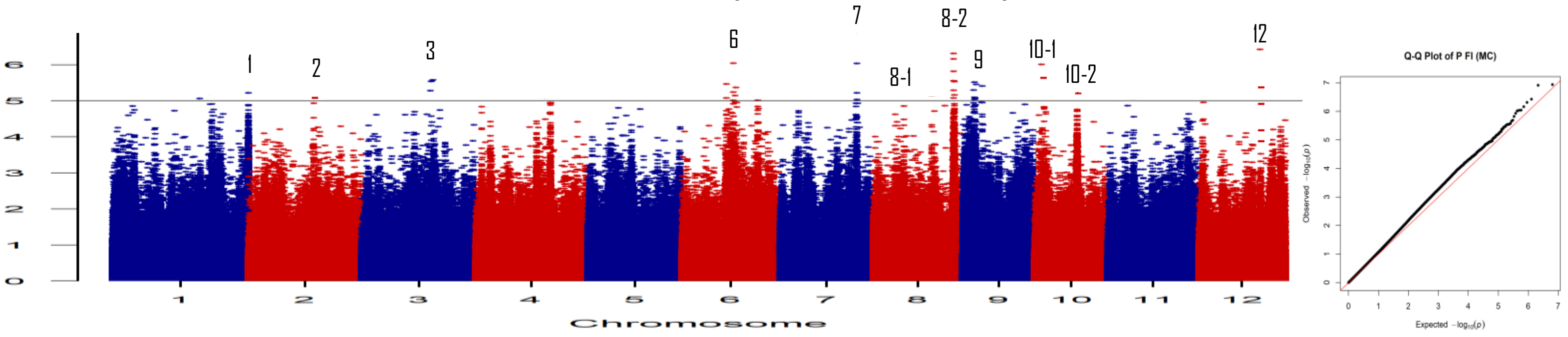

(K) Grain-S concentration – analyzed across 2 years, all Minicore

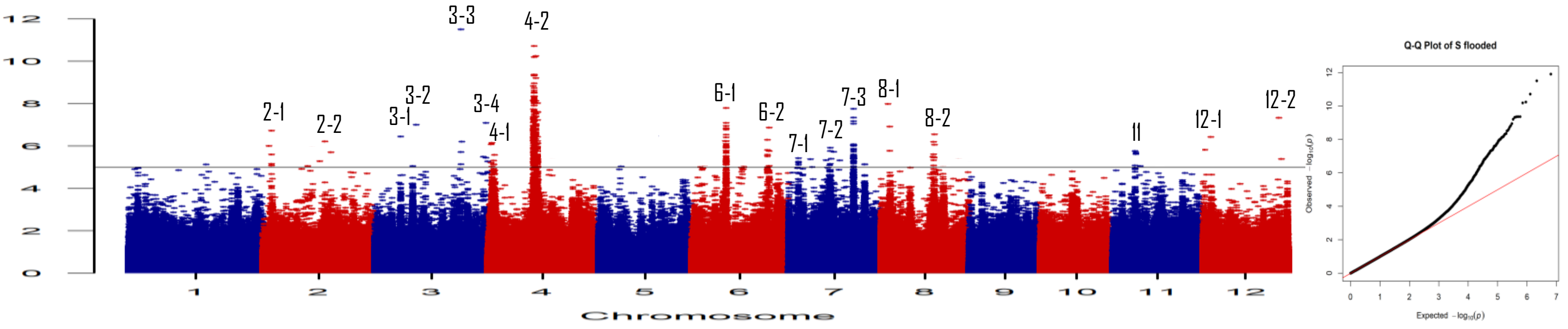

(L) Grain-Ca concentration – analyzed across 2 years, all Minicore

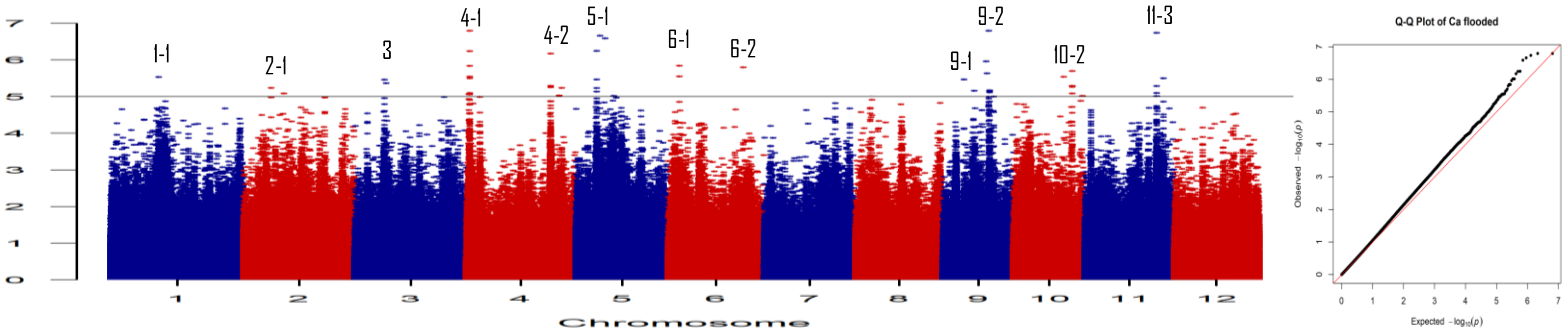

(M) Grain-Ca concentration – analyzed across 2 years, *japonica* subspecies

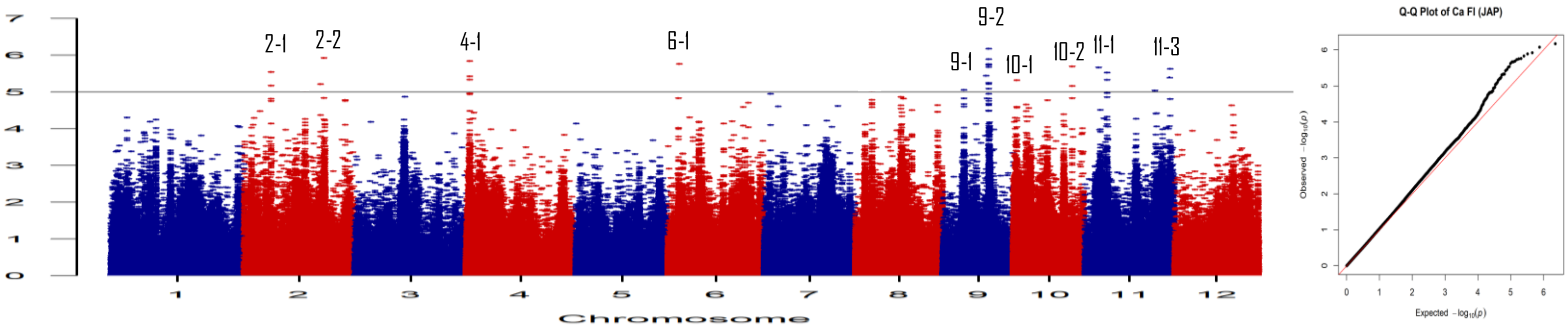

(N) Grain-Ca concentration – analyzed across 2 years, IND subpopulation

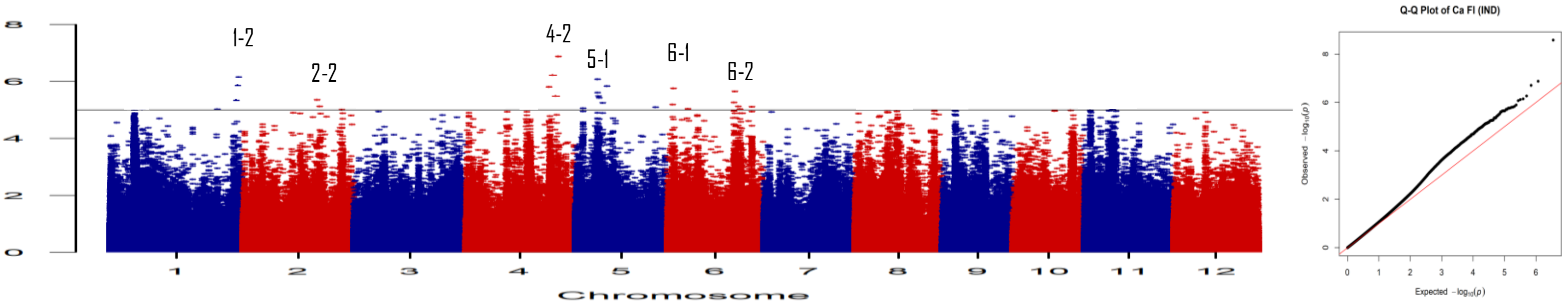

(O) Grain-Ca concentration – analyzed across 2 years, AUS subpopulation

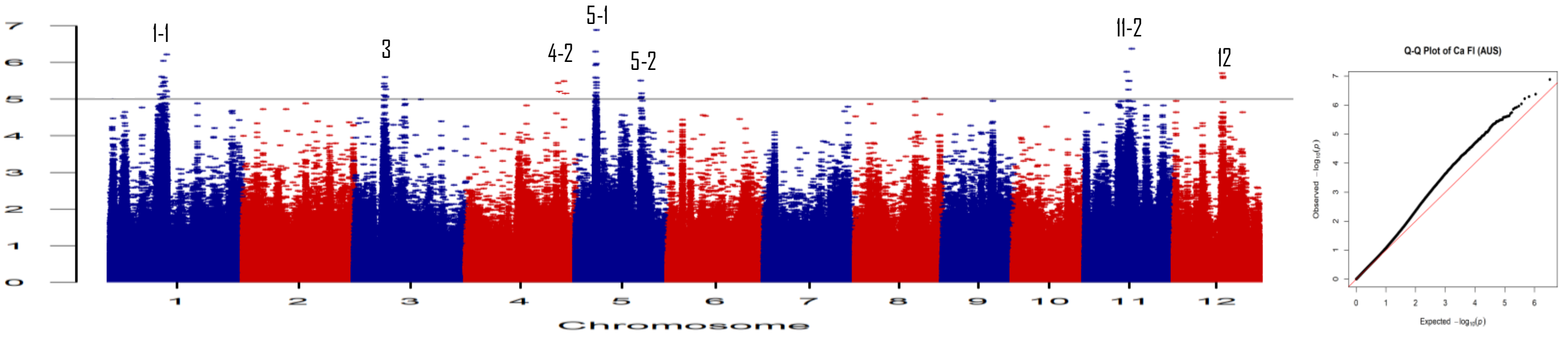

(P) Grain-Ca concentration – analyzed across 2 years, all Minicore

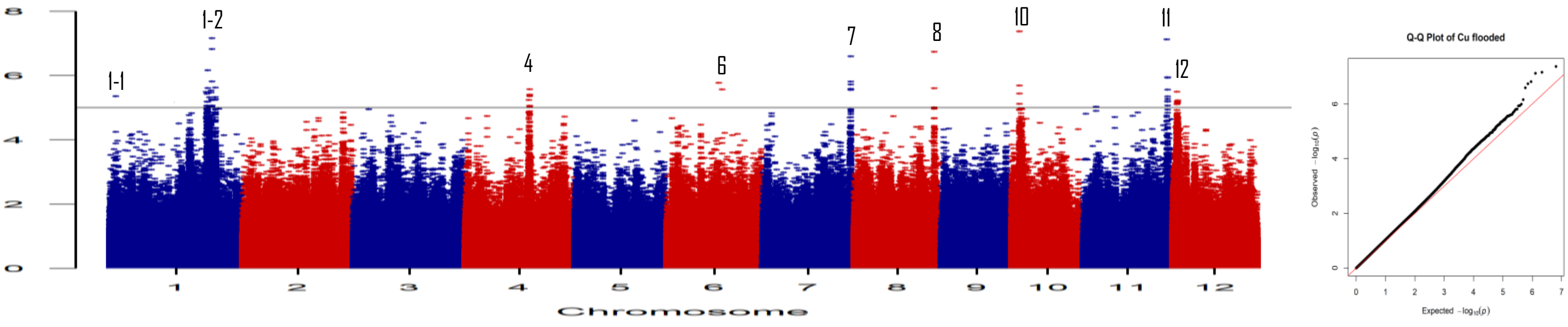

(Q) Days to heading, native soil across 2 years – All Minicore

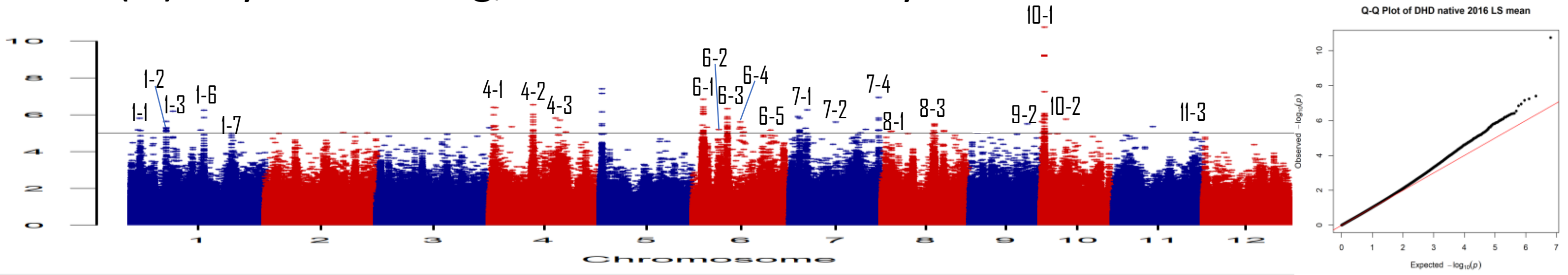

(R) Days to heading, native soil across 2 years, *indica* subspecies

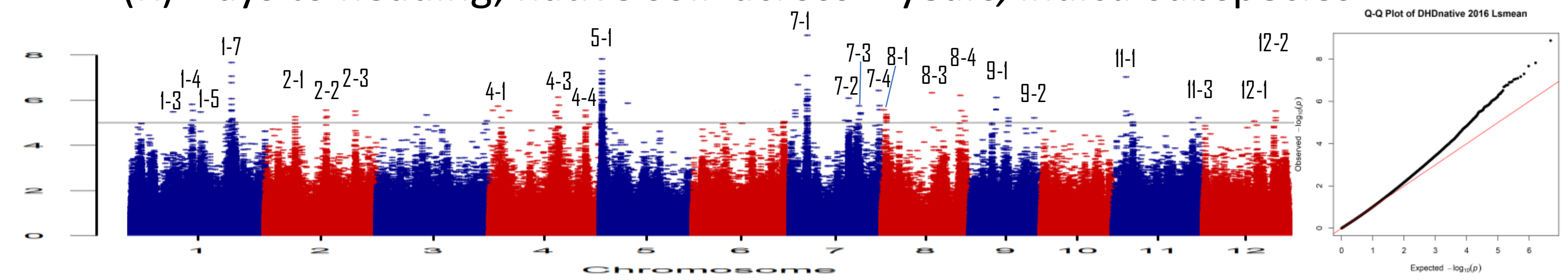

(S) Days to heading, native soil across 2 years, *japonica* subspecies

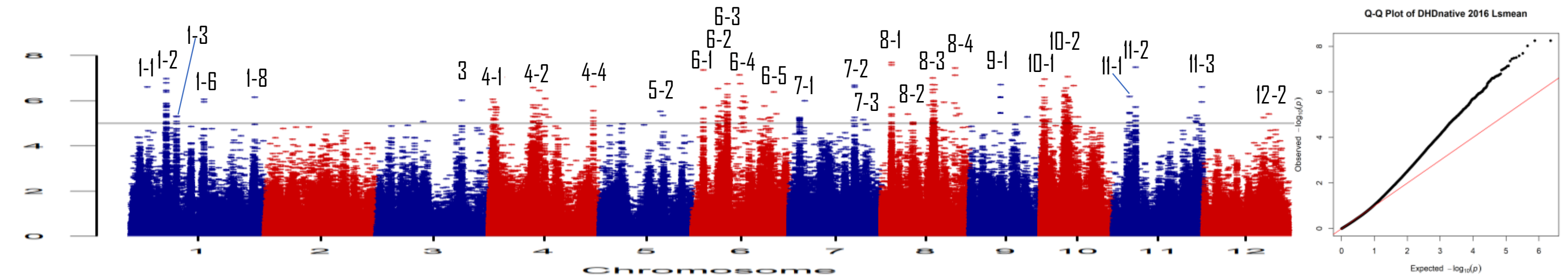

(T) Plant height, native soil across 2 years – All Minicore

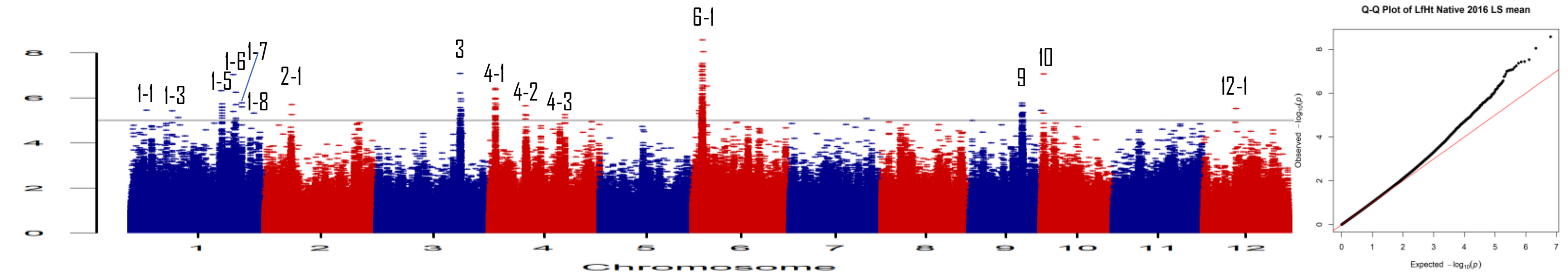

(U) Plant height, native soil across 2 years, *indica* subspecies

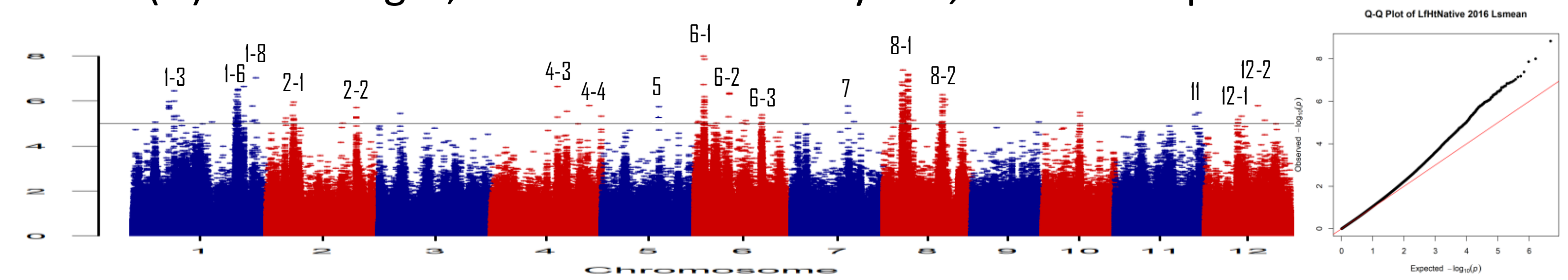

(V) Plant height, native soil across 2 years, *Japonica* subspecies

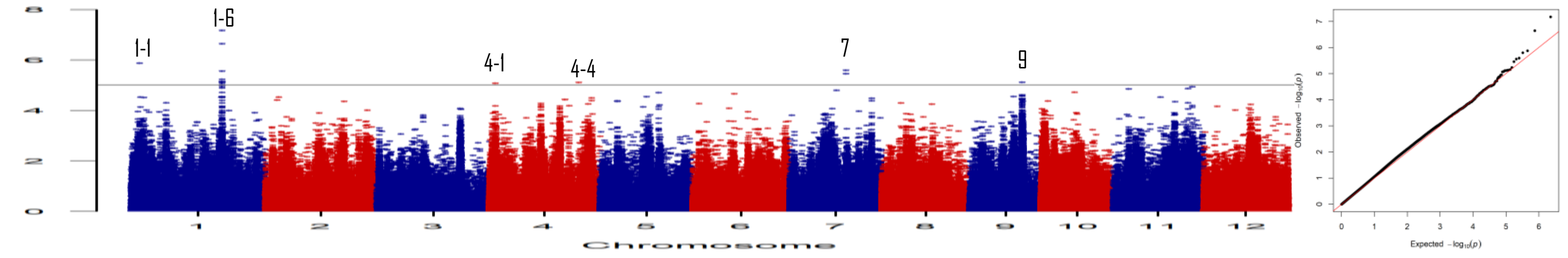

Supplement: Supplementary file 4 [file Image1.pdf]
